# Targeting ENO1 reprograms macrophage polarization to trigger antitumor immunity and improves the therapeutic effect of radiotherapy

**DOI:** 10.1038/s41419-026-08416-7

**Published:** 2026-02-02

**Authors:** Yu-Sen Lin, Hsin-Yu Chang, Wei-Ze Hong, Jhen-Yu Chen, Wei-Ching Huang, Ta-Tung Yuan, Tao-Wei Ke, Yuan-Yao Tsai, Te-Hong Chen, Ji-An Liang, Jui-I Chao, K. S. Clifford Chao, Kevin Chih-Yang Huang

**Affiliations:** 1 Department of Chest Surgery, China Medical University Hospital, Taichung, Taiwan; 2 Institute of Molecular Medicine and Bioengineering, National Yang Ming Chiao Tung University, Hsinchu, Taiwan; 3 Center of Proton Therapy, China Medical University Hospital, China Medical University, Taichung, Taiwan; 4 HuniLife Biotechnology, Taipei City, Taiwan; 5 Department of Colorectal Surgery, China Medical University Hospital, China Medical University, Taichung, Taiwan; 6 School of Chinese Medicine, China Medical University, Taichung, Taiwan; 7 Graduate Institute of Biomedical Sciences, China Medical University, Taichung, Taiwan; 8 Department of Surgery, China Medical University Hospital, Taichung, Taiwan; 9 Department of Radiation Oncology, China Medical University Hospital, China Medical University, Taichung, Taiwan; 10 Department of Biological Science and Technology, National Yang Ming Chiao Tung University, Hsinchu, Taiwan; 11 Center for Intelligent Drug Systems and Smart Bio-Devices, National Yang Ming Chiao Tung University, Hsinchu, Taiwan; 12 Department of Biomedical Imaging and Radiological Science, China Medical University, Taichung, Taiwan; 13 Translation Research Core, China Medical University Hospital, China Medical University, Taichung, Taiwan; 14 Cancer Biology and Precision Therapeutics Center, China Medical University, Taichung, Taiwan; 15 Office of Research and Development, Asia University, Taichung, Taiwan

**Keywords:** Tumour immunology, Colorectal cancer

## Abstract

Enolase 1 (ENO1) is a glycolytic enzyme involved in tumor progression that performs a variety of classical and nonclassical functions. However, the mechanism by which it promotes tumor progression is still not fully understood. Here, we revealed that TGFβ1/Smad3 signaling triggered the symmetric dimethylation of arginine (SDMA) on ENO1 by protein arginine methyltransferase 5 (PRMT5), leading to membranous ENO1 translocation. Surface ENO1 interacts with monocarboxylate transporter 4 (MCT4) for lactate secretion, which recruits M2 macrophages and promotes an immunosuppressive tumor microenvironment (TME). Targeting surface ENO1 with HuL001, a first-in-class humanized antibody, significantly reduced glycolysis, decreased extracellular lactate accumulation, reprogrammed macrophage polarization and inhibited tumor growth and distant metastasis. Moreover, targeting surface ENO1 significantly increased the therapeutic response to radiotherapy and delayed tumor regrowth by increasing antitumoral M1 macrophages and cytotoxic CD8^+^ T cells infiltration within TME. These results indicated that targeting surface ENO1 remodeled the tumor microenvironment and provided better therapeutic effects to radiotherapy in poorly immunogenic colorectal cancer (CRC) and triple-negative breast cancer (TNBC).

## Introduction

Tumors evolve to escape tumor immunosurveillance and inhibit antitumor immunity via various mechanisms during tumorigenesis and development. Immune checkpoint signaling is one of the main mechanisms for tumor immune escape [1, 2]. Currently, the clinical efficacy of immune checkpoint inhibitors (ICIs) is remarkable for the treatment of multiple cancers [3–5]. However, the clinical response to ICIs is not satisfactory in some immune-resistant tumors, such as breast cancer and colorectal cancer (CRC), due to the immunosuppressive tumor microenvironment (TME), suggesting that alternative novel immunotherapeutic strategies for cancer treatment are necessary [6].

Changes in energy metabolism are a hallmark of cancer progression [7], such as the Warburg effect, which is a well-known metabolic feature associated with highly elevated turnover of pyruvate into lactate, resulting in a limited therapeutic response and poor patient prognosis [8, 9]. The Warburg phenotype also represents a metabolic checkpoint contributing to immune evasion and a poor response to immunotherapy through the release of lactate via proton-coupled monocarboxylate transporter 4 (MCT4) [10]. Lactate transport via MCT4 results in extracellular lactate accumulation and concomitant acidification in the tumor microenvironment (TME), fostering immunosuppressive cell populations and limiting antitumor immune effector cells [8, 10]. Under these conditions, tumor-associated macrophages exhibit tumor-promoting features such as increased secretion of interleukin 6 (IL-6) and transforming growth factor β (TGFβ1). Additionally, TGFβ1 is also elicited by radiation in cancer cells to hinder the cross-priming of T cells by impairing the antigen-presenting function of dendritic cells and the functional differentiation of T cells into effectors [11]. Therefore, TGFβ1 is the most potent suppressor of the antitumor immune response to limit the therapeutic response to radiotherapy within the TME [12].

Enolase 1 (ENO 1) is a glycolytic enzyme that promotes phosphoenolpyruvate formation from 2-phospho-glycerate and then generates ATP during glycolysis [13]. ENO1 precipitates during cancer occurrence, development and metastasis in different malignancies [14–17] and enhances resistance to chemotherapy in breast cancer, pancreatic ductal adenocarcinoma (PDAC) and CRC [18–20]. ENO1 is also considered a potential immunotherapeutic target to increase dendritic cell (DC) maturation and the killing ability of cytotoxic T lymphocytes (CTLs) and natural killer (NK) cells to eradicate tumor cells [21, 22]. In addition to its critical enzymatic role in glycolysis, ENO1 can translocate from the cytosol to the cell surface and act as a plasminogen receptor to activate plasmin-dependent cancer invasion and metastasis [23–25]. Surface ENO1 overexpression is also associated with the progression of breast, lung, and PDAC [26]. Notably, blockade of surface ENO1 with anti-ENO1 antibodies has shown significant therapeutic efficacy in multiple myeloma (MM), prostate cancer, lung cancer and PDAC [24, 27–29]. Targeting surface ENO1 inhibited glycolysis and T-cell-mediated antitumor immunity against MM cytotoxicity and improved patient outcomes [22, 29], suggesting that ENO1 is a novel immunotherapeutic target for reshaping the tumor microenvironment.

In this study, we showed that ENO1 membrane translocation was regulated by TGFβ1/Smad3 signaling-mediated PRMT5 activation. PRMT5 promoted ENO1 methylation and membrane translocation to interact with MCT4 for lactate secretion. Targeting surface ENO1 with HuL001, a first-in-class humanized antibody, significantly reduced glycolysis, decreased extracellular lactate accumulation, reprogrammed macrophage polarization and inhibited tumor growth and distant metastasis. Additionally, combination treatment with HuL001 and radiotherapy significantly increased complete response and delayed tumor regrowth in CRC and triple-negative breast cancer (TNBC) animal models. We found that macrophages could be repolarized from the M2- phenotype to an antitumoral M1-like phenotype within the tumor microenvironment, resulting in a highly immunogenic TME. Therefore, the number of cytotoxic CD8^+^ T cells is significantly increased to enhance antitumor immunity and enhance the clinical benefit of radiotherapy. These similar therapeutic strategies also delayed tumor growth and reduced the risk of distant metastasis in a poorly immunogenic TNBC animal model. Taken together, these results indicate that targeting surface ENO1 can provide better therapeutic response to radiotherapy by remodeling TME in poorly immunogenic cancers.

## Materials and methods

### Cell lines, drugs and antibodies

The mouse colon cancer cell line CT26, mouse breast cancer cell line 4T1, human CRC cell lines (SW480, SW620, HCT116, LoVo and HT29) and human breast cancer cell lines (MDA-MB-468, BT-20, HS578T and SUM-159) were obtained from the ATCC. The cell lines were cultured in complete RPMI 1640 supplemented with 10% fetal bovine serum, 2 mM L-glutamine, and 1% (v/v) penicillin/streptomycin and maintained at 37 °C in 5% CO_2_. The cells were not further authenticated but were cultured for a limited number of passages. The cell lines were tested for the absence of mycoplasma contamination using PCR.

The TGFβR1 inhibitor galunisertib (HY-13226, MCE, USA) [30], Smad3 inhibitor SIS3 (HY-13013, MCE, USA) and PRMT5 inhibitor EPZ015666 (HY-12727, MCE, USA) were dissolved in DMSO to a concentration of 10 mM. Aliquots were stored at −80 °C and diluted with media before use [31, 32]. A human anti-ENO1 mAb (HuL001) is a humanized derivative of a murine parental mAb that is cross-reactive to both human and mouse ENO1 but does not bind to ENO2 or ENO3 [24].

### Western blotting analysis

Total cell lysates (30 μg) were separated using sodium dodecyl sulfate‒polyacrylamide gel electrophoresis (SDS‒PAGE, 6–12% resolving gel) and were electrophoretically transferred to PVDF membranes (GE, Amersham, UK). The membranes were blocked with 5% nonfat milk and probed with specific antibodies overnight at 4 °C. Then, horseradish peroxidase (HRP)-conjugated secondary antibodies (1:10,000, GE Healthcare, Amersham, UK) were added to the membranes, followed by detection using the Immobilon Western Chemiluminescent HRP Substrate (Millipore, MA, USA) [33]. Densitometric analysis of the western blots was performed via an ImageQuant™ LAS 4000 biomolecular imager (GE Healthcare, Amersham, UK). The digital images were processed with Adobe Photoshop 7.0 (Adobe Systems, CA, USA). Each blot was stripped using Restore Western Blot Stripping Buffer (Pierce, IO, USA) and incubated with the other antibodies. The results were assessed using ImageJ software (NIH, MD, USA). The antibodies used were as follows: anti-ENO1 (ab155102, Abcam, Cambridge), anti-PRMT5 (ab109451, Abcam), anti-phospho-Smad2/3 (AP1343, ABclonal), anti-SMDA (PA5-116813, Thermo Fisher), anti-MCT4 (ab308528, Abcam) and anti-Na/K ATPase (ab254025, Abcam).

### RNA sequencing (RNA-Seq) and data analysis

Total RNA was extracted from the cell lines using TRIzol reagent. Messenger RNA was extracted from the total RNA and cut into short fragments of ~200 bases for use as templates for cDNA synthesis. The cDNAs were subsequently used to establish a cDNA library using PCR amplification and sequenced via the Illumina HiSeqTM 2500 platform. Clean reads were obtained by trimming the adaptor sequences from the raw reads, and these reads were subsequently subjected to further transcript annotation and calculation with the fragments per kilobase per million reads (FPKM) method. Differentially expressed genes (DEGs) were identified with the DESeq software package. The Benjamini–Hochberg false discovery rate was used to correct the *p* values, with the significance level set at 0.05.

### qRT‒PCR

Total RNA was extracted from the cell lines with TRIzol (Invitrogen, CA, USA), quantified by measuring the absorbance at 260 nm, and then reverse-transcribed into cDNA using iScript™ Reverse Transcription Supermix (Bio-Rad, CA, USA) according to the manufacturer’s instructions. Primers were designed using the Primer Design Tool (NCBI, USA) according to sequence information from the NCBI database. qRT‒PCR was performed in a final reaction volume of 20 μl with iQ™ SYBR® Green Supermix (Bio-Rad, CA, USA) using the CFX96 Touch Real-Time PCR Detection System (Bio-Rad) [34, 35]. All the reactions were performed in triplicate for each sample, and GAPDH was used as a reference gene for normalization. The relative gene expression levels were calculated using the 2 − ΔΔCt method. Gene expression levels were compared via t tests [30, 36].

### Immunohistochemistry

The following antibodies were used in this study: anti-ENO1 (ab227978, Abcam), anti-CD68 (ab303565, Abcam) and anti-CD163 (ab182422, Abcam). The tissue slides were deparaffinized, incubated with 3% H_2_O_2_ in water for 10 min to decrease endogenous peroxidase activity, and subjected to heat-mediated antigen retrieval with antigen unmasking solution (H3300, Vector Laboratories, Burlingame, CA) [37]. Tissue sections (3 µm thick) were stained with the HRP-conjugated avidin-biotin complex (ABC) from the Vectastain Elite ABC Kit (Vector Laboratories, Burlingame, CA) and DAB chromogen (Vector Laboratories) and counterstained with hematoxylin. The immune cells were positive when they were detected in the tumor-infiltrating immune cells and were evaluated using a microscope (OLYMPUS BX53, Tokyo, Japan). For the detection of TILs, the tissue was viewed at 40× magnification, and the area with the highest density of CD68^+^ and CD163^+^ immune cells among the malignant cells was counted at 400× magnification (number of immune cells/high-power field). The average number of tumor-infiltrating immune cells in five high-power fields was included in the evaluation [38, 39].

### Treatment of tumor-bearing animals with anti-ENO1 antibodies (HuL001)

CT26 cells (2.5×10^5^ cells/mouse) were suspended in 100 μl of 50% Matrigel matrix and subcutaneously injected into the left back of each BALB/c mouse. On Day 11, when the tumor size reached 100 mm^3^, the animals were randomly assigned to 2 groups and received anti-ENO1 antibodies (HuL001, 20 mg/kg, intraperitoneal injection) on the indicated days. The tumor volume was measured every 3 days, and the mice were sacrificed on Day 32. The tumor volumes were calculated according to the following formula: (width^2^ × length)/2. The mice were sacrificed at the end of the experiments, and tumor tissues from representative mice were collected for lysis, subjected to western blot analysis and subjected to immunohistochemistry [40, 41].

For the metastatic CRC model, CT26 cells (2.5 × 10^5^ cells/mouse) were suspended in 100 μl of PBS and intravenously (IV) injected into the lateral tail vein of each BALB/c mouse. On Day 11, the animals were randomly assigned to 2 groups receiving anti-ENO1 antibodies (HuL001, 40 mg/kg, intraperitoneal injection) on the indicated days, and the mice were sacrificed on Day 32. Lung tissues from representative mice were collected for immunohistochemistry.

For the TNBC animal model, 4T1 cells (5 × 10^4^ cells/mouse) were suspended in 100 μl of PBS and intravenously (IV) injected into the lateral tail vein of each BALB/c mouse. The animals were randomly assigned to 2 groups receiving anti-ENO1 antibodies (HuL001, 40 mg/kg, intraperitoneal injection) on the indicated days, and the mice were sacrificed on Day 32. Lung tissues from representative mice were collected for immunohistochemistry.

### Evaluation of the immunomodulatory effect of HuL001 in response to radiotherapy in tumor-bearing animals

CT26 cells (2.5 × 10^5^ cells/mouse) were suspended in 100 μl of 50% Matrigel matrix and subcutaneously injected into the left leg of each BALB/c mouse. On Day 4, the animals were randomly assigned to 4 groups receiving HuL001 (40 mg/kg, intraperitoneal injection) alone or in combination or not with local radiotherapy (5 Gy) on the indicated days. The tumor volume was measured every 3 days, and the mice were sacrificed on Day 30. The tumor volumes were calculated according to the following formula: (width^2^ × length)/2. The mice were sacrificed at the end of the experiments, and tumor tissues were collected for flow cytometry analysis and immunofluorescence staining.

4T1 cells (5 × 10^4^ cells/mouse) were suspended in 100 μl of 50% Matrigel matrix and subcutaneously injected into the left leg of each BALB/c mouse. On Day 4, the animals were randomly assigned to 4 groups receiving HuL001 (40 mg/kg, intraperitoneal injection) alone or in combination or not with local radiotherapy (8 Gy × 2) on the indicated days. The tumor volume was measured every 3 days, and the mice were sacrificed on Day 40. The tumor volumes were calculated according to the following formula: (width^2^ × length)/2. The mice were sacrificed at the end of the experiments, and tumor tissues were collected for flow cytometry analysis and immunofluorescence staining.

### Flow cytometry analysis of immune cell profiles

Tumors were dissected from the mice, weighed, and then placed in Petri dishes containing blank RPMI media at room temperature to prevent dehydration [42]. Tumors were minced into small pieces (1–2 mm) with a beaver blade, filtered through a 70-μm strainer, centrifuged, and then resuspended in blank RPMI media. Thereafter, the cell suspensions were layered over Ficoll-Paque media and centrifuged at 1025 ×*g* for 20 min. The layer of mononuclear cells was transferred to a conical tube, 20 ml of complete RPMI media was added, the mixture was gently mixed, and the sample was centrifuged at 650 ×*g* for 10 min twice. Finally, the supernatant was removed, and the TILs were resuspended in complete RPMI media [38, 43, 44].

Then, the TILs were resuspended in 500 μL of staining buffer (2% BSA and 0.1% NaN_3_ in PBS). The cells were stained with different antibodies against combinations of surface markers: (1) CD4 and CD8 T cells: ViaDyeRed, CD45-BV785 (E-AB-F1136UD, Elabscience, Texas, USA), CD3-PE-Fir700, CD4-PE/Cy7, CD8a-BV570 (E-AB-F1104UF, Elabscience, Texas, USA), CD127-BV711, CD25, IFNγ-PE and GzmB-AF647; (2) Tumor-associated macrophages: ViaDyeRed, CD3-PE-Fir700, CD19-PE-Fir700, CD45-BV785, CD11b-BV421, F4/80-PE, CD11c-AF488, CD206-APC. For intracellular staining, the TILs were fixed and permeabilized with a Foxp3/transcription factor staining buffer set (eBioscience, Thermo Fisher, CA, USA) after cell surface staining. The cells were then stained with IFNγ and GzmB for 45 min. The samples were washed twice with Perm Wash Buffer and then analyzed with Cytek Aurora (Cytek® Biosciences, CA, USA). These antibodies were purchased from Cytek® Biosciences.

### Construction of the tissue microarray (TMA)

Tissue microarrays were constructed from 156 surgical tumor tissues and adjacent normal tissues from stage III colorectal cancer patients [45]. The tumor cell areas were evaluated and marked on hematoxylin and eosin (H&E)-stained slides, and the corresponding area on the paraffin block (donor block) was then identified and moved by an AutoTiss 10 C system (EverBio Technology Inc., Taipei, Taiwan) to a recipient block. Each punch was 2 mm in diameter, and a maximum of 60 punches were placed on a single block to generate sections with a microtome [32, 45].

### Statistical analysis

Two-tailed Student’s t tests were used to determine the statistical significance of the differences between the means. Tumor sizes were compared between the groups using the Mann‒Whitney U test. Survival analyses were plotted using Kaplan‒Meier curves. A *p* value of <0.05 was considered to indicate statistical significance. All the statistical tests were performed using SPSS Manager software.

## Results

### High surface ENO1 is associated with a risk of distant metastasis and poor survival outcomes in multiple malignancies

**Fig. 1:**
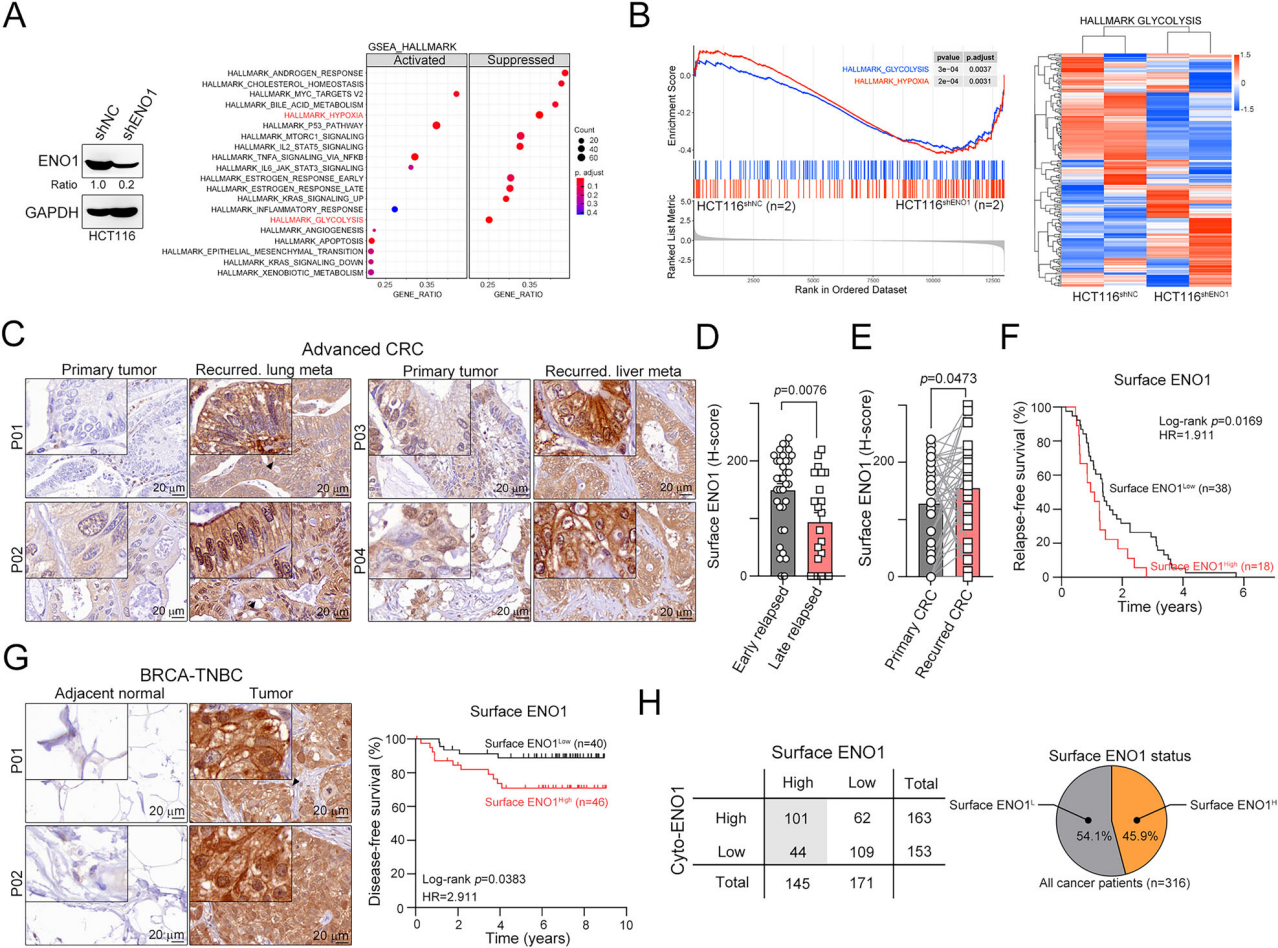
High surface expression of ENO1 was associated with poor survival outcomes in advanced CRC patients and TNBC patients. **A** HCT116 cells were infected with lentivirus carrying shRNA against ENO1 or negative control (NC) for 24 hr. The cells were selected with puromycin for three days. The knockdown efficacy of ENO1 was evaluated by immunoblotting. The differential transcriptome was analyzed by RNA-seq. **B** The glycolysis pathway was analyzed by GSEA and a heatmap (*n* = 2). **C** The level of surface ENO1 in primary and distant recurrent tumors from matched paired advanced CRC patients was analyzed by immunohistochemistry (*n* = 56). **D** Advanced CRC patients were stratified into early and late recurrence subgroups to analyze the association with surface ENO1 by immunohistochemistry (H-score, *n* = 56 and *p* = 0.0076). **E** The level of surface ENO1 in primary and distant recurrent tumors was examined by immunohistochemistry (H-score, *n* = 56 and *p* = 0.0473). **F** Advanced CRC patients with high surface ENO1 were associated with poor relapse-free survival (RFS, *n* = 56, log-rank *p* = 0.0169). **G** The level of surface ENO1 in primary tumors from BRCA-TNBC patients was analyzed by immunohistochemistry (*n* = 86). BRCA-TNBC patients with high surface ENO1 were associated with poor disease-free survival (DFS, *n* = 86, log-rank *p* = 0.0383). **H** High expression of surface ENO1 was detected in ~45.9% of cancer patients, including advanced CRC (*n* = 56), BRCA-TNBC (*n* = 86) and lung cancer (*n* = 174) patients.

ENO1 is a key glycolytic enzyme that is responsible for the transformation of 2‑phosphoglycerate into phosphoenolpyruvate [13]. Indeed, we found that the knockdown of ENO1 significantly inhibited hypoxia and the glycolysis signaling pathway (GSEA_HALLMARK_HYPOXIA and GSEA_HALLMARK_GLYCOLYSIS; Fig. 1A, B). In addition to its role as a glycolytic enzyme, ENO1 is expressed on the cell surface and acts as a plasminogen receptor to activate plasmin-dependent cancer invasion and metastasis [24, 25]. Therefore, to assess the correlation between surface ENO1 expression and advanced CRC and TNBC patients, we measured the levels of cytoplasmic and surface ENO1 in advanced CRC (*n* = 56, Fig. 1C) and BRCA-TNBC patients (*n* = 86, Fig. 1F). We found that high surface ENO1 protein levels were correlated with a high risk of tumor relapse (Fig. 1D, *p* = 0.0076, *n* = 56). Moreover, elevated surface ENO1 was detected in distant recurrent CRC compared with primary CRC tumors (Fig. 1E, *p* = 0.0473, *n* = 56). Advanced CRC patients with high surface ENO1 were associated with poor survival outcomes (Fig. 1F, log-rank *p* = 0.0169, hazard ratio (HR) = 1.911, *n* = 56). High surface ENO1 was also found in TNBC patients and was associated with worse survival outcomes (Fig. 1G, log-rank *p* = 0.0383, hazard ratio (HR) = 2.911, *n* = 86). Similar results were found in lung cancer patients (Fig. S1A, log-rank *p* = 0.0371, hazard ratio (HR) = 1.891, *n* = 174) and The Cancer Genome Atlas (TCGA)-lung adenocarcinoma (Fig. S1B, log-rank *p* = 0.0008, hazard ratio (HR) = 1.746, *n* = 500). Overall, we found ~45.9% (145/316) of patients had surface ENO1 expression, suggesting that surface ENO1 is also associated with the risk of metastasis, recurrence and survival in CRC, TNBC and lung cancer patients.

### TGFβ1 promotes ENO1 membrane translocation via the TGFβR1/Smad3/PRMT5 pathway

**Fig. 2:**
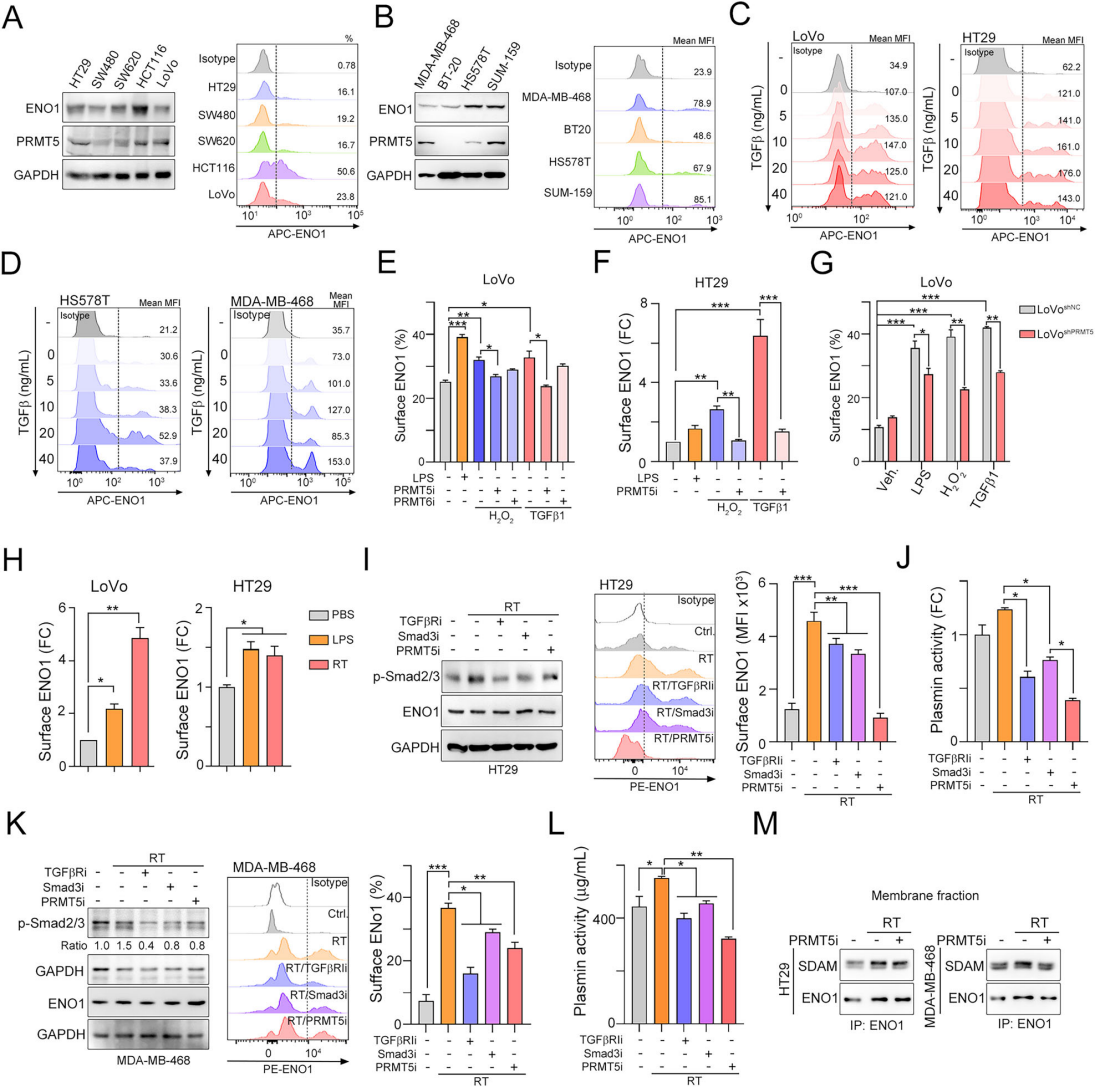
Radiotherapy promotes TGFβ1/Smad3 signaling for surface ENO1 translocation via PRMT5. **A** The expression of total and surface ENO1 in human CRC cell lines was evaluated by immunoblotting and flow cytometry. **B** The expression of total and surface ENO1 in human BRCA cell lines was evaluated by immunoblotting and flow cytometry. **C** HT29 and LoVo cells were treated with different doses of recombinant human TGFβ1 protein (rhTGFβ1) for 24 hr, after which surface ENO1 expression was analyzed via flow cytometry. **D** HS578T and MDA-MB-468 cells were treated with different doses of the rhTGFβ1 protein for 24 hr, after which the surface ENO1 level was analyzed via flow cytometry. **E** LoVo cells were treated with LPS (1 μg/mL), rhTGFβ1 protein (10 ng/mL) or H_2_O_2_ (50 μM) in combination with a PRMT5 inhibitor (AMG-193, 1 μM) or a PRMT6 inhibitor (EPZ020411 hydrochloride, 1 μM) for 24 hr. The surface ENO1 level was analyzed via flow cytometry. *p < 0.05, **p < 0.01 and ***p < 0.001. One-Way ANOVA test (*n* = 3). **F** HT29 cells were treated with rhTGFβ1 protein in combination with a PRMT5 inhibitor (1 μM) for 24 hr. The surface ENO1 expression was analyzed via flow cytometry. **p < 0.01 and ***p < 0.001. One-Way ANOVA test (*n* = 3). **G** LoVo^shNC^ and LoVo^shPRMT5^ cells were treated with LPS (1 μg/mL), rhTGFβ1 protein (10 ng/mL) or H_2_O_2_ (50 μM) for 24 hr. The surface level of ENO1 was examined by flow cytometry. *p < 0.05, **p < 0.01 and ***p < 0.001. One-Way ANOVA test (*n* = 3). **H** HT29 and LoVo cells were treated with LPS (1 μg/mL) and RT (5 Gy) for 24 hr. The surface level of ENO1 was examined by flow cytometry. *p < 0.05 and **p < 0.01. One-Way ANOVA test (*n* = 3). **I** HT29 cells were treated with RT (5 Gy) in combination with a TGFβR1 inhibitor (1 μM), a Smad3 inhibitor (1 μM), or a PRMT5 inhibitor (1 μM) for 24 hr. The surface level of ENO1 was examined by flow cytometry. ***p < 0.001. One-Way ANOVA test (*n* = 3). **J** HT29 cells were treated with RT (5 Gy) in combination with a TGFβR1 inhibitor (1 μM), a Smad3 inhibitor (1 μM), or a PRMT5 inhibitor (1 μM) for 24 hr. The level of plasmin activity was examined via an ELISA kit. *p < 0.05. One-Way ANOVA test (*n* = 3). **K** MDA-MB-468 cells were treated with RT (5 Gy) in combination with a TGFβR1 inhibitor (1 μM), a Smad3 inhibitor (1 μM), or a PRMT5 inhibitor (1 μM) for 24 hr. The surface level of ENO1 was examined by flow cytometry. *p < 0.05, **p < 0.01 and ***p < 0.001. One-Way ANOVA test (*n* = 3). **L** MDA-MB-468 cells were treated with RT (5 Gy) in combination with a TGFβR1 inhibitor (1 μM), a Smad3 inhibitor (1 μM), or a PRMT5 inhibitor (1 μM) for 24 hr. The level of plasmin activity was examined via an ELISA kit. *p < 0.05, and **p < 0.01. One-Way ANOVA test (*n* = 3). **M** HT29 cells were treated with RT (5 Gy) in combination with a PRMT5 inhibitor (1 μM) for 24 hr. The level of surface-methylated ENO1 was evaluated by immunoprecipitation and immunoblotting.

In accordance with the results of immunohistochemical analysis, surface ENO1 was detected to different extents in CRC and TNBC cell lines (Fig. 2A, B). To determine the detailed mechanisms of ENO1 membrane translocation, we treated CRC (LoVo and HT29) and TNBC (HS578T and MDA-MB-468) cell lines with proinflammatory cytokine TGFβ1 and ROS inducer H_2_O_2_, which have been reported to induce ENO1 membrane translocation [26]. Indeed, we found that TGFβ1 significantly upregulated surface ENO1 in CRC and TNBC cell lines (Fig. 2C, D). Previous studies have reported that protein arginine methyltransferase 5 (PRMT5) and PRMT6 mediate ENO1 methylation for glycolysis and membrane translocation [46, 47]. By treating with a PRMT5 inhibitor (EPZ015666) or PRMT6 inhibitor (EPZ020411) in combination with TGFβ1 or H_2_O_2_, we found that PRMT5 inhibition significantly decreased the level of surface ENO1 induced by TGFβ1 or H_2_O_2_ in LoVo cells (Fig. 2E). However, treatment with the PRMT6 inhibitor had little influence on the level of surface ENO1 (Fig. 2E). We also found that PRMT5 inhibition significantly decreased the level of surface ENO1 induced by TGFβ1 or H_2_O_2_ in HT29 cells (Fig. 2F). PRMT5 knockdown markedly decreased the level of surface ENO1 induced by TGFβ1 and H_2_O_2_ in LoVo cells (Fig. 2G). Since radiotherapy promotes TGFβ1 and ROS accumulation [11], we assumed that radiotherapy (RT) may upregulate surface ENO1 expression. Indeed, we found that RT triggered surface ENO1 accumulation (Fig. 2H). To confirm that TGFβ1-mediated surface ENO1 accumulates via PRMT5 after RT, we combined a TGFβR1 inhibitor (galunisertib), a Smad3 inhibitor (SIS3) and a PRMT5 inhibitor (EPZ015666) to evaluate the level of surface ENO1 after RT. We found that blockade of TGFβR1/Smad3 significantly decreased the level of surface ENO1, and direct inhibition of PRMT5 markedly decreased surface ENO1 after RT (Fig. 2I). Since surface ENO1 functions as a plasminogen receptor for plasmin production [27], we also detected plasmin activity in the conditioned medium (Fig. 2J). We found that inhibition of TGFβR1 and PRMT5 resulted in lower levels of plasmin activity after RT (Fig. 2J). Similarly, we found that inhibition of TGFβR1 and PRMT5 resulted in lower levels of surface ENO1 and plasmin activity after RT (Fig. 2K, L). Moreover, the level of methylation of ENO1 (symmetric dimethylation of arginine, SDMA) was significantly decreased by the PRMT5 inhibitor after RT (Fig. 2M), suggesting that the methylation of ENO1 by TGFβ1/Smad3/PRMT5 might promote ENO1 membrane translocation.

### Targeting surface ENO1 with the first-in-class ENO1 mAb HuL001 decreased tumor growth and distant metastasis and inhibited glycolytic processes

**Fig. 3:**
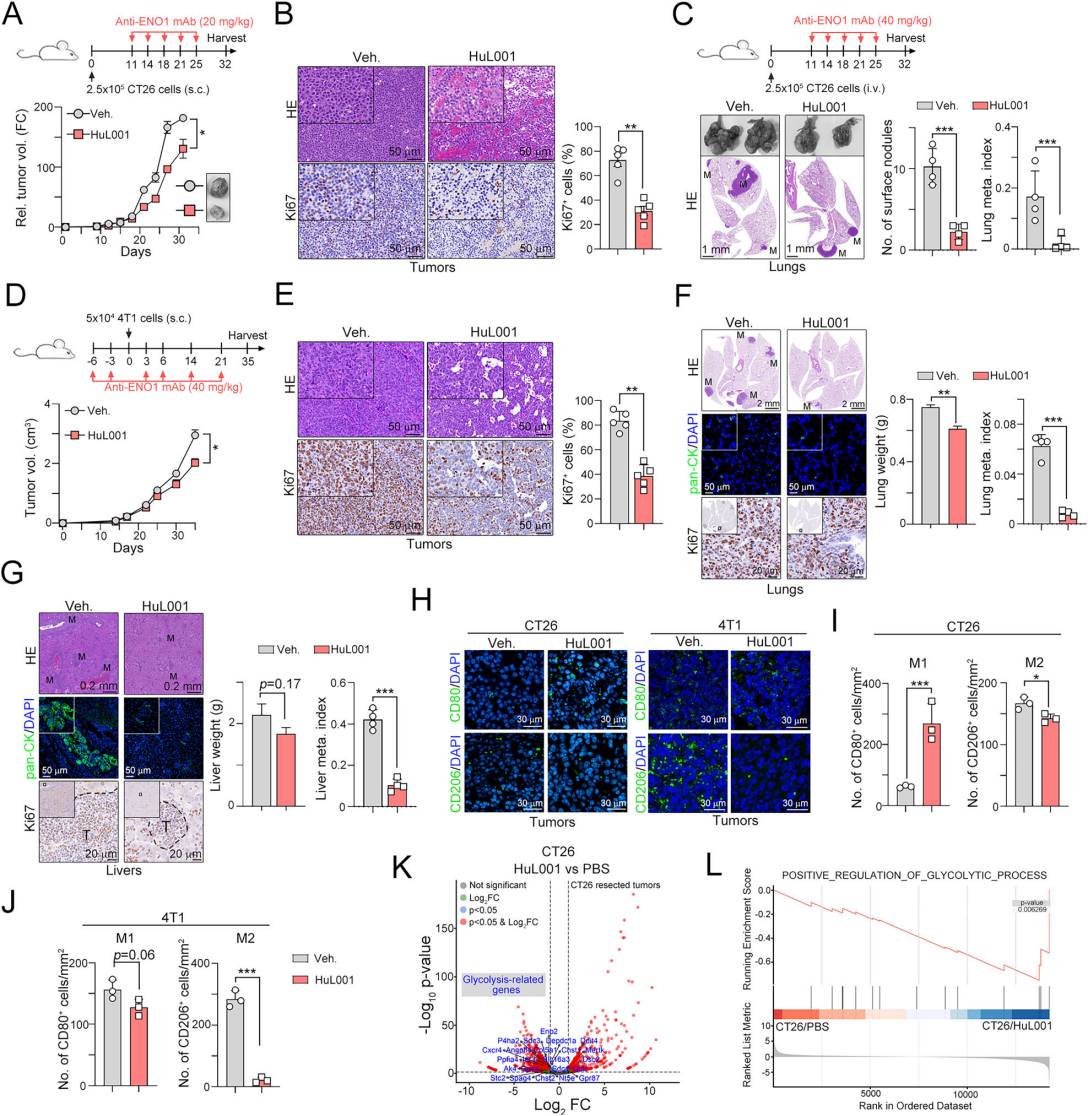
Radiotherapy promotes TGFβ1/Smad3 signaling for surface ENO1 translocation via PRMT5. **A** A total of 2.5 × 10^5^ CT26 cells were subcutaneously injected into BALB/c mice for 11 days and then intraperitoneally administered anti-ENO1 antibodies (HuL001, 20 mg/kg) five times at three-day intervals (*n* = 5). The tumor volume was recorded every three days. *p < 0.05. Two-Way ANOVA test (*n* = 5). **B** The resected tumors from the CT26 tumor-bearing mice were analyzed by immunohistochemical analysis. **p < 0.01. Unpaired t test (*n* = 5). **C** A total of 2.5 × 10^5^ CT26 cells were intravenously injected into BALB/c mice for 11 days and then intraperitoneally administered anti-ENO1 antibodies (HuL001, 40 mg/kg) five times at three-day intervals (*n* = 5). The tumor volume was recorded every three days. The resected lung tissues were analyzed by HE staining. ***p < 0.001. Unpaired t test (*n* = 5). **D** 4T1 cells (5 × 105) were intravenously injected into BALB/c mice, which were then intraperitoneally administered anti-ENO1 antibodies (HuL001, 40 mg/kg) on the indicated days (n = 5). The tumor volume was recorded every three days. *p < 0.05. Two-Way ANOVA test (*n* = 5). **E** The resected tumors from 4T1-bearing mice were analyzed by HE staining. **p < 0.01. Unpaired t test (*n* = 5). **F** The resected lung tissues from 4T1-bearing mice were analyzed by HE staining, immunofluorescence staining and immunohistochemical analysis. **p < 0.01 and ***p < 0.001. Unpaired t test (*n* = 5). **G** The resected liver tissues from 4T1-bearing mice were analyzed by HE staining, immunofluorescence staining and immunohistochemical analysis. ***p < 0.001. Unpaired t test (*n* = 5). **H** The density of M1 (CD80^+^) and M2 (CD206^+^) macrophages in resected tumors from CT26- and 4T1-bearing mice was evaluated by immunofluorescence staining (*n* = 3). **I** The quantification of M1 (CD80^+^) and M2 (CD206^+^) macrophages in resected tumors from CT26-bearing mice. *p < 0.05 and ***p < 0.001. Unpaired t test (*n* = 5). **J** The quantification of M1 (CD80^+^) and M2 (CD206^+^) macrophages in resected tumors from 4T1-bearing mice. *p < 0.05 and ***p < 0.001. Unpaired t test (*n* = 5). **K** The DEGs between HuL001- and Veh-treated resected CT26 tumors are shown in a volcano plot (*n* = 2). **L** Compared with those in Veh.-treated resected tumors, the number of glycolysis-related gene sets significantly decreased in HuL001-treated resected tumors (*n* = 2).

Targeting surface ENO1 has been shown to inhibit tumor invasiveness and metastasis by decreasing plasmin levels [24, 29]. Therefore, we evaluated the therapeutic effects of the first-in-class ENO1 mAb HuL001 [24]. After five cycles of treatment with HuL001, we observed significant suppression of tumor growth in a mouse model of synergistic colon cancer (Fig. 3A). The density of the proliferating cell marker Ki67 was significantly reduced after HuL001 treatment in a mouse CRC model (Fig. 3B). Moreover, distant lung metastatic tumor nodules were also reduced after HuL001 administration in a mouse CRC model (Fig. 3C). Consistent with these results, blockade of ENO1 by HuL001 significantly delayed tumor growth in a mouse TNBC model (Fig. 3D, E). Moreover, targeting ENO1 also decreased spontaneous metastatic tumor formation in the lungs and liver (Fig. 3F, G), suggesting that surface ENO1 participated in tumor progression and metastasis. Interestingly, we found that the density of tumor-associated macrophages was markedly decreased in tumors resected from mouse CRC and TNBC models (Fig. 3H–J). To further identify the potential mechanism of surface ENO1 in cancer progression, we analyzed the transcriptomic profile of resected tumors after HuL001 treatment. We found that the glycolytic signaling pathway was suppressed after HuL001 treatment, suggesting that surface ENO1 also promoted glycolytic metabolism for cancer metastasis (Fig. 3K, L). Moreover, we found that the immune population, especially the macrophage population, significantly changed, according to the assessment of known cell type markers in the xCell dataset after HuL001 treatment (Fig. S2). The number of immunosuppressive M2 macrophages was reduced after HuL001 treatment. Taken together, these results indicate that targeting ENO1 may suppress glycolytic metabolism to reshape the TME by decreasing the number of immunosuppressive M2 macrophages.

**Fig. 4:**
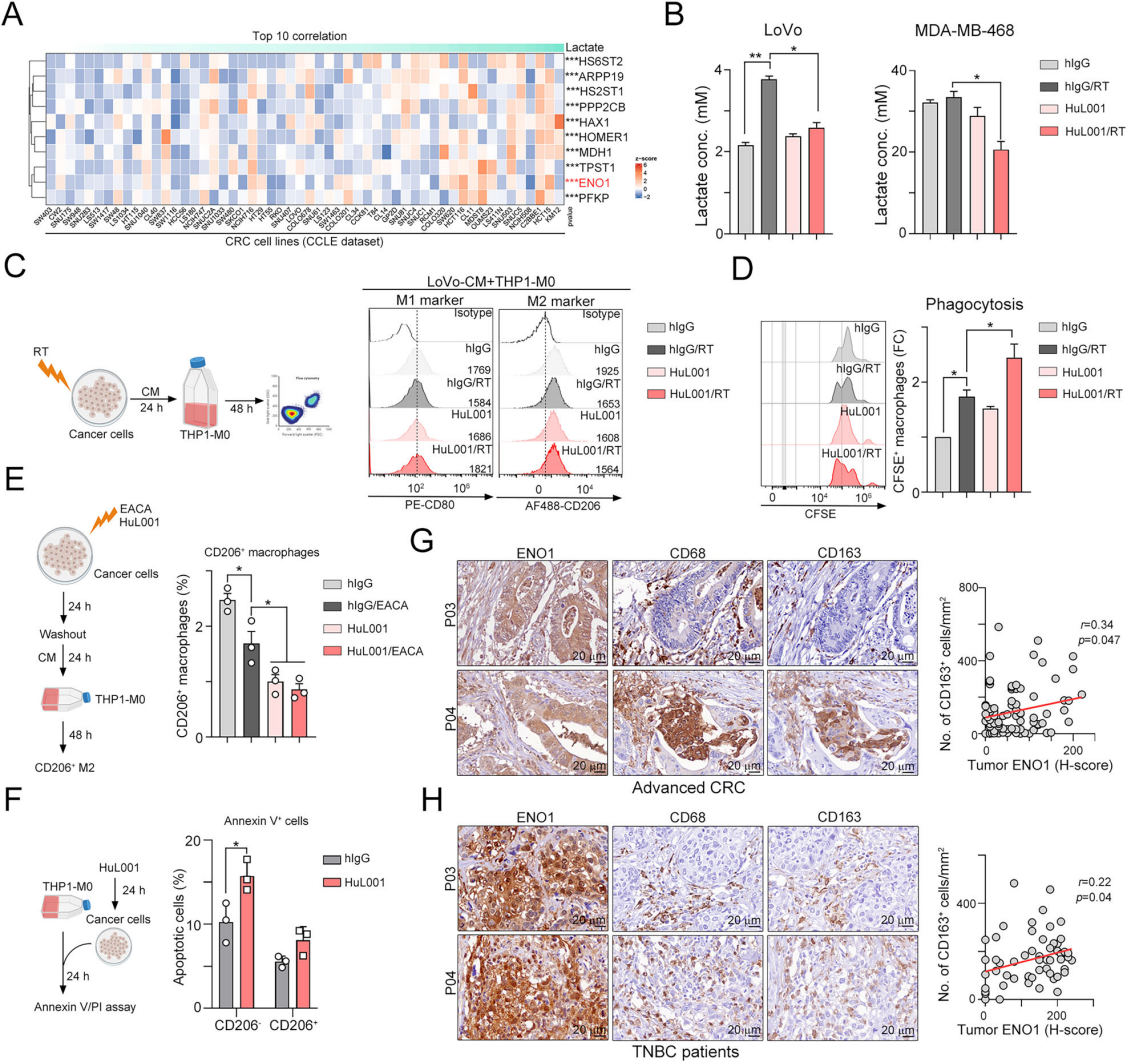
Surface ENO1 was associated with lactase release and correlated with the infiltration of macrophages in CRC and TNBC patients. **A** The correlation between lactate concentration and *ENO1* mRNA expression was evaluated in human CRC cell lines (CCLE database). **B** LoVo and MDA-MB-468 cells were irradiated (5 Gy) and treated with anti-ENO1 (HuL001, 100 μg/mL) for 24 hr. The level of lactate in the conditioned medium (CM) was examined via an ELISA kit. *p < 0.05 and **p < 0.01. One-Way ANOVA test (*n* = 3). **C** LoVo cells were irradiated (5 Gy) and treated with anti-ENO1 (HuL001, 100 μg/mL) for 24 hr. The conditioned medium was collected to treat the THP-1-M0 cells for 24 hr. The levels of M1 (CD80^+^) and M2 (CD206^+^) markers were evaluated by flow cytometry. **D** The CM-treated THP-1-M0 cells were cocultured with LoVo cells for 18 hr, and phagocytosis was evaluated via flow cytometry. *p < 0.05. One-Way ANOVA test (*n* = 3). **E** LoVo cells were treated with plasmin inhibitor (EACA, 10 μM) or HuL001 (100 μg/mL) for 24 hr. The conditioned medium was harvested after 24 hr, and incubated with THP-1-M0 cells for 24 hr. The percentage of CD206^+^ M2 was evaluated by flow cytometry. *p < 0.05. One-Way ANOVA test (*n* = 3). **F** THP-1-M0 cells were cocultured with LoVo cells for 24 hr, and treated with HuL001 (100 μg/mL) for 24 hr. The apoptotic cells were evaluated via flow cytometry. *p < 0.05. One-Way ANOVA test (*n* = 3). **G** The correlation of surface ENO1 (CD80^+^) and M2 (CD206^+^) markers in advanced CRC patients was analyzed by immunohistochemistry (*n* = 56). Spearman correlation analysis (*p* = 0.01 and *r* = 0.337). **H** The correlation of surface ENO1 (CD80^+^) and M2 (CD206^+^) markers in BRCA-TNBC patients was analyzed by immunohistochemistry (*n* = 86). Pearson correlation analysis (*p* = 0.047 and *r* = 0.215).

Lactate is the end product of aerobic glycolysis and is transported across the plasma membrane by MCT4 to maintain the flow of glycolysis [10]. Indeed, we found that *ENO1* mRNA expression was positively correlated with the level of extracellular lactate in Cancer Cell Line Encyclopedia database (CCLE, Fig. 4A). Therefore, we assumed that blockade of surface ENO1 may inhibit extracellular lactate accumulation. Indeed, we found that targeting surface ENO1 with HuL001 significantly decreased the extracellular lactate concentration after radiotherapy in CRC and TNBC cells (Fig. 4B, Fig. S3A). Given that lactate is known to impact macrophage polarization within the TME [48], we next evaluated whether targeting ENO1 triggers metabolic reprogramming in macrophages within the TIME. Therefore, we differentiated THP-1 monocytes into M0 macrophages and incubated them with conditioned medium (CM) collected from HuL001-treated cells for 48 h. Compared with RT alone, RT and HuL001 significantly upregulated the level of M1 marker CD80 (Fig. 4C). In contrast, the M2 marker CD206 was decreased by RT and HuL001 treatment compared with RT alone (Fig. 4C). Additionally, the phagocytotic ability also increased after treatment with CM from the RT and HuL001 groups (Fig. 4D). These results suggest that surface ENO1 modulates the immunosuppressive tumor microenvironment by lactate-driven macrophage polarization. Since surface ENO1 acts as a plasminogen receptor to promotes macrophage recruitment during inflammation [24, 29], we then evaluate whether ENO1-mediated lactate secretion for macrophage polarization was independent of plasmin. Therefore, we individually treated with plasmin inhibitor 6-aminocaproic acid (EACA) [49] and HuL001 to cancer cells, and then collected these conditioned medium for THP1-M0 cells. As shown in Fig. 4E, we found that the percentage of CD206^+^ M2 was significantly decreased by EACA and HuL001, especially by HuL001-treated CM. Moreover, dual treatment with EACA and HuL001 did not significantly decreased the percentage of CD206^+^ M2, suggesting that targeting surface ENO1 may not only inhibit plasmin-mediated macrophage recruitment/polarization but inhibit lactate-mediated macrophage polarization. Furthermore, we did not observe significant CD206^+^ M2 cell death after treatment with HuL001 (Fig. 4F), suggesting that targeting surface ENO1 did not directly eradicate macrophage. But we found that HuL001 also directly increased colorectal cancer cell death (Fig. 4F). For clinical relevance, we evaluate the correlation between macrophage and surface ENO1 in CRC and BRCA-TNBC patients. Indeed, we found that the level of surface ENO1 was positively correlated with the density of macrophages (CD68^+^M1 and CD163^+^M2) within the TME in CRC patients, especially CD163^+^M2 macrophages (Fig. 4G and Fig. S3B, *p* = 0.02, *r* = 0.39). A positive correlation between surface ENO1 and macrophages was also found in BRCA-TNBC patients (Fig. 4H). Taken together, these results suggest that surface ENO1 modulates the immunosuppressive tumor microenvironment by lactate-driven tumor-associated macrophage polarization to promote cancer progression and metastasis.

### Surface ENO1 directly interacts with MCT4 to stabilize and release lactate

**Fig. 5:**
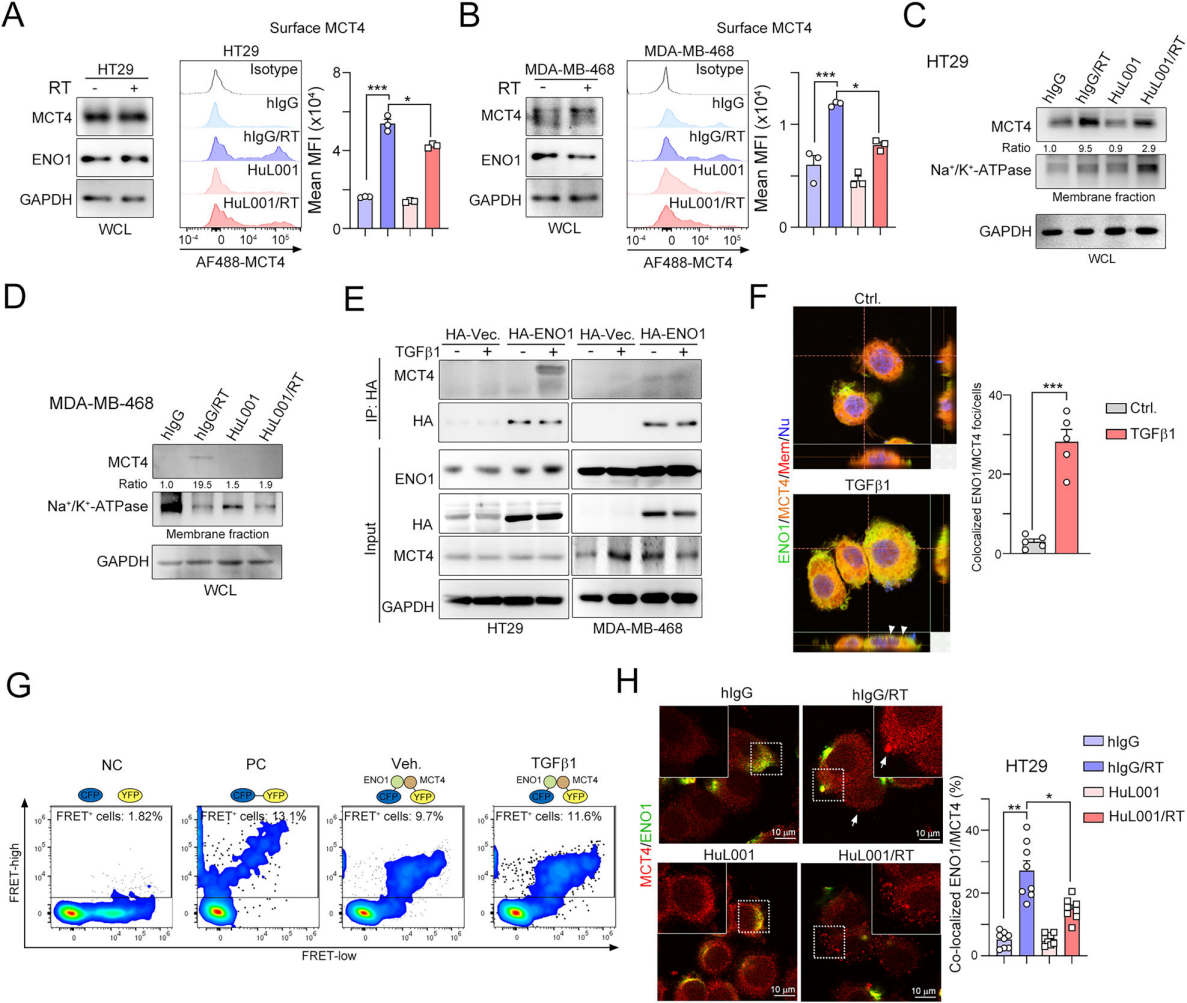
Surface ENO1 directly interacts with MCT4 to release lactate. **A** HT29 cells were treated with RT (5 Gy) in combination with anti-ENO1 antibodies (HuL001) for 24 hr. The surface level of MCT4 was detected by flow cytometry (*n* = 3). WCL whole cell lysate. *p < 0.05 and ***p < 0.001. One-Way ANOVA test (*n* = 3). **B** MDA-MB-468 cells were treated with RT (5 Gy) in combination with anti-ENO1 antibodies (HuL001) for 24 hr. The surface level of MCT4 was detected by flow cytometry (*n* = 3). *p < 0.05 and ***p < 0.001. One-Way ANOVA test (*n* = 3). **C** HT29 cells were treated with RT (5 Gy) in combination with anti-ENO1 antibodies (HuL001) for 24 hr. The membrane fraction was isolated for immunoblotting. **D** MDA-MB-468 cells were treated with RT (5 Gy) in combination with anti-ENO1 antibodies (HuL001) for 24 hr. The membrane fraction was isolated for immunoblotting. **E** HT29 cells were transfected with HA-vector (Vec.) and HA-ENO1 for 24 hr and then treated with rhTGFβ1 protein (10 ng/mL) for 24 hr. The cell lysates were harvested for immunoprecipitation and immunoblotting. **F** HT29 cells were treated with rhTGFβ1 protein (10 ng/mL) for 24 hr. The colocalization of MCT4 and ENO1 was analyzed by confocal microscopy. ***p < 0.001. Unpaired t test (*n* = 3). **G** HT29 cells were individually transfected with CFP, YFP, CFP-YGP or CFP-ENO1/YFP-MCT4 for 24 hr and then treated with rhTGFβ1 protein (10 ng/mL) for 24 hr. FRET was analyzed by flow cytometry. **H** HT29 cells were treated with RT (5 Gy) in combination with anti-ENO1 antibodies (HuL001) for 24 hr and then analyzed by confocal microscopy. The quantification of colocalized membranous MCT4 and ENO1 is shown. *p < 0.05 and **p < 0.01. One-Way ANOVA test (*n* = 3).

It has been reported that MCT4-mediated lactate secretion can polarize M2 macrophages and suppress T-cell-mediated cytotoxicity [10]. Since targeting surface ENO1 with HuL001 decreased lactate secretion, we examined whether the level of MCT4 was reduced if surface ENO1 was targeted by HuL001 via flow cytometry. The level of surface MCT4 was increased after radiotherapy (Fig. 5A). However, after treatment with HuL001, the level of surface MCT4 was markedly reduced after radiotherapy in CRC and TNBC cells (Fig. 5A, B). By subcellular fractionation, we found that the level of surface MCT4 was significantly decreased by HuL001 after radiotherapy in HT29 and MDA-MB-468 cells (Fig. 5C, D).

We then examined whether surface ENO1 interacted with MCT4 to stabilize it for lactate secretion. By immunoprecipitation, we found that ENO1 directly interacted with MCT4 after TGFβ1 treatment (Fig. 5E). Furthermore, the EN01 and MCT4 was also co-localized on the surface after TGFβ1 treatment (Fig. 5F). To confirm the direct interaction between ENO1 and MCT4, we generated ENO1-CFP and MCT4-YFP constructs, and transfected into HT29 cells to analyze their interaction by a FACS-based FRET assay (Fig. 5G). To establish a FACS-based FRET assay, we first analyzed 293 T cells expressing CFP and YFP controls individually, in combination or as a fusion protein (Fig. 5G). We gated living cells according to forward and sideward scatter (FSC/SSC) and adjusted photomultiplier tube (PMT) voltages and compensated for CFP and YFP to specifically assess FRET in double-positive cells (Fig. 5G). We plotted FRET versus CFP to determine the number of FRET-positive cells. As shown in Fig. 5G, when the cells were cotransfected with the ENO1-CFP and MCT4-YFP constructs, 9.7% FRET was detected. Moreover, the number of FRET-positive cells increased after TGFβ1 treatment (11.6%), suggesting that ENO1 directly interacted with MCT4 to release lactate. Consistently, we found that ENO1 colocalized with MCT4 at the plasma membrane (Fig. 5H). The degree of colocalization was markedly increased by RT. However, upon treatment with HuL001, colocalization was markedly reduced at the plasma membrane (Fig. 5H). Taken together, these results indicate that surface ENO1 directly interacts with MCT4 to stabilize and release lactate.

### The targeting of surface ENO1 by HuL001 significantly changed macrophage polarization to increase the therapeutic response to radiotherapy

**Fig. 6:**
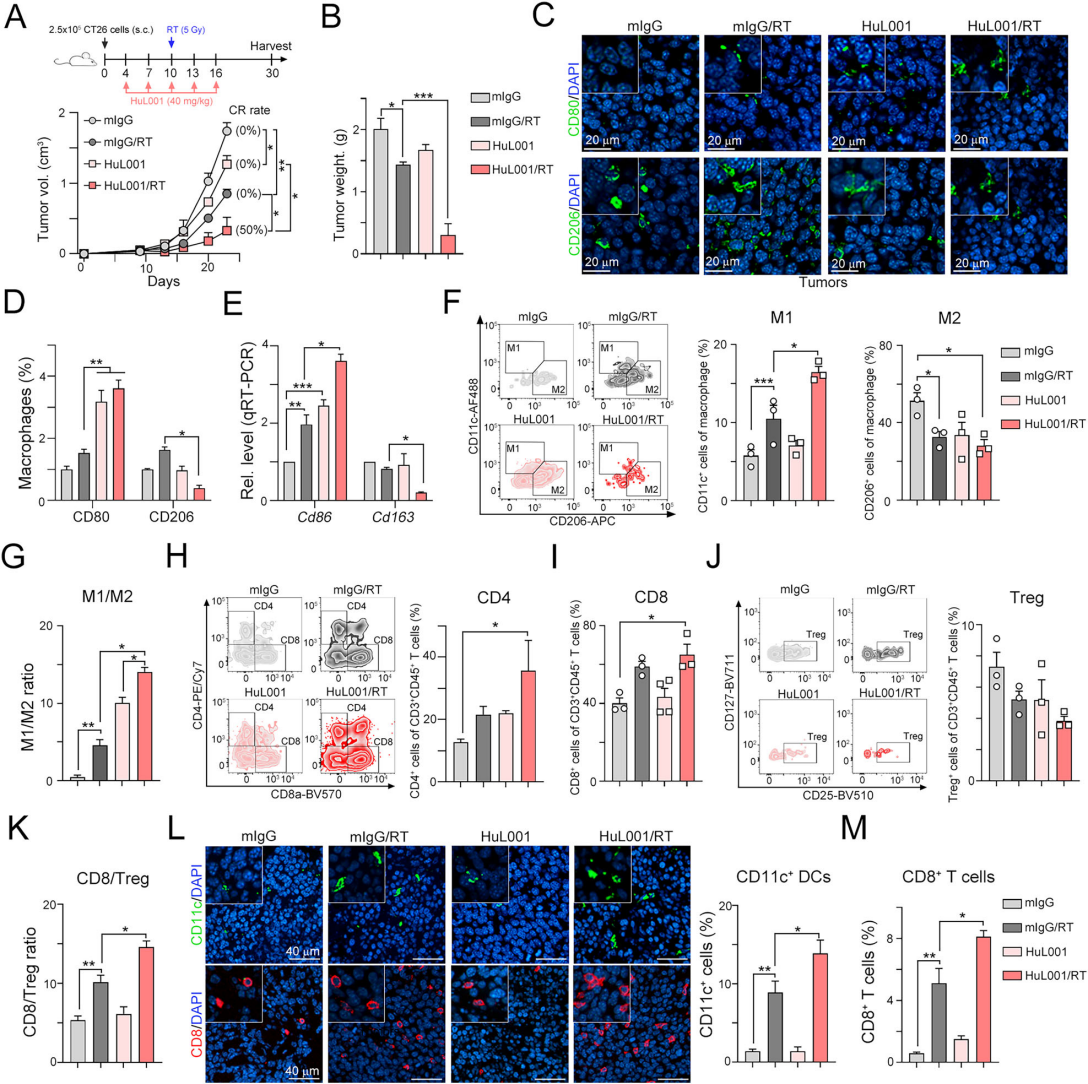
Blockade of ENO1 significantly enhances the response to radiotherapy and triggers antitumor immunity by reshaping the tumor microenvironment in CRC. **A** A total of 2.5 × 10^5^ CT26 cells were subcutaneously injected into the left legs of BALB/c mice for 4 days and then intraperitoneally administered anti-ENO1 antibodies (HuL001, 20 mg/kg) five times at three-day intervals. Local radiotherapy was given on Day 10. The tumor volume was recorded every three days. *p < 0.05 and **p < 0.01. Two-Way ANOVA test (*n* = 4). **B** The resected tumors were weighed on Day 30. *p < 0.05 and ***p < 0.001. One-Way ANOVA test (*n* = 3). **C** The densities of M1 (CD80^+^) and M2 (CD206^+^) macrophages in resected tumors were analyzed by immunofluorescence staining. **D** The quantification of M1 (CD80^+^) and M2 (CD206^+^) macrophages in resected tumors. *p < 0.05 and **p < 0.01. One-Way ANOVA test (*n* = 3). **E** The mRNA levels of *Cd86* and *Cd163* in resected tumors were analyzed by qRT‒PCR (*n* = 3). *p < 0.05, **p < 0.01 and ***p < 0.001. One-Way ANOVA test (*n* = 3). **F** The frequencies of M1 (CD11c^+^CD11b^+^F4/80^+^CD45^+^7AAD^-^CD3^-^CD19^-^) and M2 (CD206^+^CD11b^+^F4/80^+^CD45^+^7AAD^-^CD3^-^CD19^-^) tumor-infiltrating macrophages were analyzed by flow cytometry (*n* = 3-4). *p < 0.05 and ***p < 0.001. One-Way ANOVA test. **G** The quantification of the M1/M2 ratio is shown (*n* = 3). *p < 0.05 and **p < 0.01. One-Way ANOVA test (*n* = 3). **H** The frequencies of CD4 (CD4^+^CD3^+^CD45^+^7AAD^-^) and CD8 (CD8^+^CD3^+^CD45^+^7AAD^-^) tumor-infiltrating T cells were analyzed by flow cytometry (*n* = 3-4). *p < 0.05. One-Way ANOVA test. **I** The quantification of CD8^+^ TILs is shown. *p < 0.05. One-Way ANOVA test (*n* = 3). **J** The frequency of regulatory CD4 T cells (CD25^+^CD127^-^CD4^+^CD3^+^CD45^+^7AAD^-^) was analyzed by flow cytometry (*n* = 3-4). **K** The quantification of the CD8/Treg ratio is shown. *p < 0.05 and **p < 0.01. One-Way ANOVA test (*n* = 3). **L** The densities of dendritic cells (CD11c^+^) and cytotoxic T cells (CD8^+^) in resected tumors were analyzed by immunofluorescence staining. The density of CD11c^+^ DCs is shown. *p < 0.05 and **p < 0.01. One-Way ANOVA test (*n* = 3). **M** The density of CD8^+^ TILs is shown. *p < 0.05 and **p < 0.01. One-Way ANOVA test (*n* = 3).

Since targeting surface ENO1 can modulate the immunosuppressive tumor microenvironment by lactate-driven tumor-associated macrophage polarization, we evaluated whether HuL001 can increase the therapeutic efficacy of radiotherapy (Fig. 6A). A total of 2.5 ×10^5^ CT26 cells were subcutaneously injected into the left legs of syngeneic BALB/c mice, which received HuL001 (40 mg/kg, i.p.) and local radiotherapy (5 Gy) on the indicated days. As shown in Fig. 6A, we found that tumor growth was clearly delayed by HuL001 and RT ( ~ 70% tumor growth inhibition, TGI). Additionally, 50% of the mice achieved complete response (CR). Similarly, the tumor weight was significantly decreased by HuL001 and RT (Fig. 6B). To further understand the changes in the proportion of macrophages in the resected tumors, we evaluated the density of M1 (CD80) and M2 (CD206) macrophages. As shown in Fig. 6C, D, we found that the number of M1 macrophages was increased and that the number of M2 macrophages was decreased by the combination of HuL001 and RT. Consistent with these results, the mRNA level of M1 (*Cd86*) was increased in the resected tumors from the HuL001/RT group (Fig. 6E).

To further understand the immune cell profile within the tumor microenvironment, we isolated immune cells from resected tumors and analyzed them via flow cytometry. The gating strategies are shown in Fig. S4. We found that the frequency of M1 macrophages (CD11c^+^CD11b^+^F4/80^+^CD45^+^ live macrophages) significantly increased after RT (Fig. 6F and Fig. S4A). Moreover, when HuL001 was combined with RT, the density of M1 increased compared with that of RT alone. In contrast, we found that the frequency of M2 macrophages (CD206^+^CD11b^+^F4/80^+^CD45^+^ live macrophages) decreased after RT. Although the frequency of M2 macrophages was not lower in the HuL001/RT group than in the RT alone group (Fig. 6F), the M1/M2 ratio was significantly greater in the HuL001/RT group than in the other groups (Fig. 6G). Furthermore, we found that the density of CD4^+^ T cells (CD4^+^CD3^+^CD45^+^ live T cells) and CD8^+^ T cells (CD8^+^CD3^+^CD45^+^ live T cells) markedly increased in the HuL001/RT group (Fig. 6H, I and Fig. S4B). The number of tumor-infiltrating regulatory T cells (Tregs, CD25^+^CD127^-^CD4^+^CD3^+^CD45^+^ live T cells) also tended to decrease (Fig. 6J). Notably, the ratio of CD8^+^ T cells/Tregs was greater in the HuL001/RT group than in the other groups (Fig. 6K). Similarly, we found that the density of tumor-infiltrating CD11c^+^ DCs and CD8^+^ T cells was markedly greater in the HuL001/RT group than in the other groups by immunofluorescent analysis (Fig. 6L, M). Taken together, these results indicate that targeting surface ENO1 may reshape the tumor microenvironment by promoting macrophage polarization to increase antitumor immunity and the therapeutic response to radiotherapy in a CRC model.

**Fig. 7:**
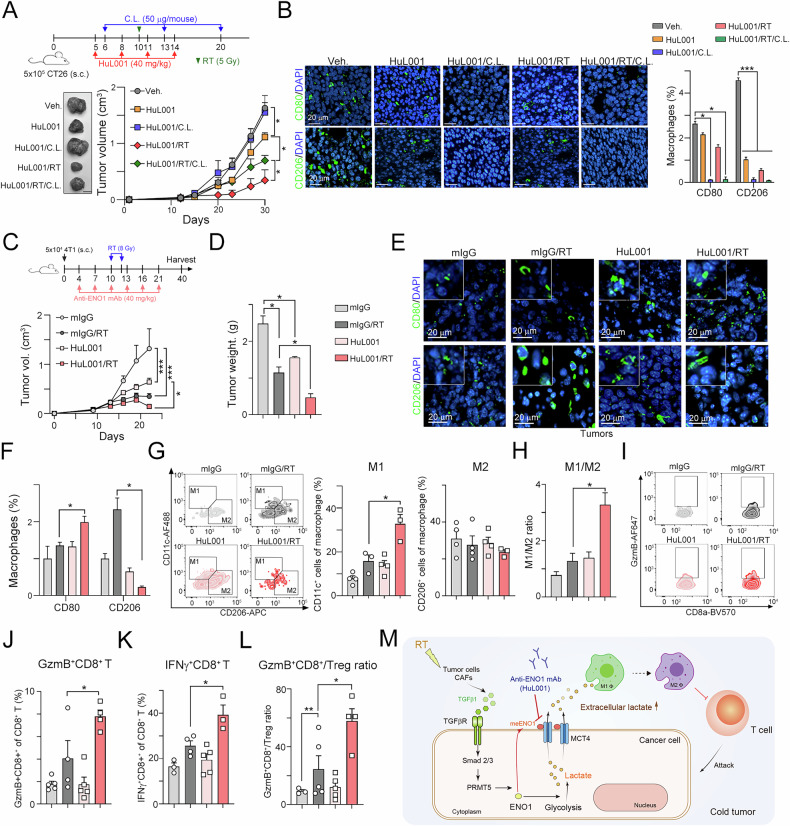
Blockade of ENO1 significantly enhanced the response to radiotherapy and triggered antitumor immunity in a TNBC animal model. **A** A total of 5 × 10^5^ CT26 cells were subcutaneously injected into the left legs of BALB/c mice for 5 days and then intraperitoneally administered with anti-ENO1 antibodies (HuL001, 40 mg/kg) or clodronate liposomes (50 μL/mouse) on the indicated days (*n* = 5). Local radiotherapy was given on Day 10. The tumor volume was recorded every three days. *p < 0.05. Two-Way ANOVA test (*n* = 4). **B** The densities of M1 (CD80^+^) and M2 (CD206^+^) macrophages in resected tumors were analyzed by immunofluorescence staining. The quantification of M1 (CD80^+^) and M2 (CD206^+^) macrophages in resected tumors. *p < 0.05 and ***p < 0.001. One-Way ANOVA test (*n* = 3). **C** 4T1 cells (5 ×10^4^) were subcutaneously injected into the left legs of BALB/c mice for 4 days and then intraperitoneally administered anti-ENO1 antibodies (HuL001, 20 mg/kg) six times on the indicated days (*n* = 5). Local radiotherapy was given on Days 10 and 12. The tumor volume was recorded every three days. *p < 0.05 and ***p < 0.001. Two-Way ANOVA test (*n* = 4). **D** The resected tumors were weighed on Day 40. *p < 0.05. One-Way ANOVA test (*n* = 4). **E** The densities of M1 (CD80^+^) and M2 (CD206^+^) macrophages in resected tumors were analyzed by immunofluorescence staining. **F** The quantification of M1 (CD80^+^) and M2 (CD206^+^) macrophages in resected tumors. *p < 0.05. One-Way ANOVA test (*n* = 3). **G** The frequencies of M1 (CD11c^+^CD11b^+^F4/80^+^CD45^+^7AAD^-^CD3^-^CD19^-^) and M2 (CD1206^+^CD11b^+^F4/80^+^CD45^+^7AAD^-^CD3^-^CD19^-^) tumor-infiltrating macrophages were analyzed by flow cytometry. *p < 0.05. One-Way ANOVA test (*n* = 3-4). **H** The quantification of the M1/M2 ratio is shown. *p < 0.05. One-Way ANOVA test (*n* = 3). **I** A representative image of flow cytometric analysis of GzmB^+^ (GzmB^hi^CD8^+^CD3^+^CD45^+^7AAD^-^) T cells. **J** The frequency of GzmB^+^ (GzmB^hi^CD8^+^CD3^+^CD45^+^7AAD^-^) T cells was analyzed by flow cytometry. *p < 0.05. One-Way ANOVA test (*n* = 3-4). **K** The density of GzmB^+^ (GzmB^hi^CD8^+^CD3^+^CD45^+^7AAD^-^) T cells is shown. *p < 0.05. One-Way ANOVA test (*n* = 3-4). **L** The density of IFNγ^+^ (IFNγ^hi^CD8^+^CD3^+^CD45^+^7AAD^-^) T cells is shown (*n* = 3-4). *p < 0.05 and **p < 0.01. One-Way ANOVA test (*n* = 3-4). **M** The proposed mechanism of TGFβ1/TGFβR/Smad3/PRMT5-mediated ENO1 translocation for lactate release via MCT4.

To further demonstrate the critical role of macrophage in HuL001 treatment, we depleted macrophage with low dose of clodronate-liposome (C.L.) [32] to evaluate the therapeutic efficacy of HuL001 in mouse colon cancer model (Fig. 7A). We found that treated with HuL001 decreased ~20% tumor volume (Fig. 7A). Combined with single dose RT and HuL001 significantly decreased ~80% tumor volume. However, treatment with C.L. significantly diminished the therapeutic efficacy of HuL001. The therapeutic efficacy of RT and HuL001 was also attenuated (Fig. 7A). We also found that the density of CD80^+^ M1 and CD206^+^ M2 was significantly depleted in C.L.-treated subgroups (Fig. 7B), suggesting that suggesting that targeting surface ENO1 remarkably increased the therapeutic response to RT was mainly dependent on the macrophage polarization to remodel TME.

To further confirm that targeting surface ENO1 remodeled the TME by promoting macrophage polarization in breast cancer, we used a similar therapeutic regimen in a 4T1-based syngeneic TNBC mouse model. 4T1 cells (5 ×10^4^) were subcutaneously injected into the left legs of syngeneic BALB/c mice, which received HuL001 (40 mg/kg, i.p.) and local radiotherapy (8 Gy) on the indicated days. We found that tumor growth was markedly delayed after HuL001 or RT monotherapy treatment (Fig. 7C). Moreover, the combination of HuL001 and RT had an extremely strong therapeutic effect on tumor growth (Fig. 7C). The weights of the resected tumors were also lower in the HuL001/RT group than in the other groups (Fig. 7D). Moreover, we found that combination treatment with HuL001 and RT clearly increased the density of CD80 + M1 macrophages and decreased the frequency of CD206 + M2 macrophages in the resected tumors (Fig. 7E, F).

By immune cell profile analysis via flow cytometry, we found that the frequency of M1 macrophages (CD11c^+^CD11b^+^F4/80^+^CD45^+^ live macrophages) was markedly increased in the HuL001/RT group (Fig. 7G). The density of M2 macrophage (CD206^+^CD11b^+^F4/80^+^CD45^+^ live macrophages) infiltration was slightly decreased in the HuL001/RT group (Fig. 7G). The M1/M2 ratio was significantly greater in the HuL001/RT group than in the other groups (Fig. 7H). Moreover, the numbers of functional cytotoxic GzmB^+^CD8^+^ T cells (GzmB^+^CD8^+^CD3^+^CD45^+^ live T cells) and IFNγ^+^CD8^+^ T cells (IFNγ^+^CD8^+^CD3^+^CD45^+^ live T cells) were markedly greater in the HuL001/RT group than in the other groups (Fig. 7I–K). There was no significant change in Tregs. However, the ratio of GzmB^+^CD8^+^ T/Treg cells was markedly greater in the HuL001/RT group than in the other groups (Fig. 7L), suggesting that HuL001 promoted antitumor immunity via macrophage repolarization to enhance the therapeutic response to RT. Taken together, these results indicate that targeting surface ENO1 can reshape the tumor microenvironment to increase antitumor immunity to increase the therapeutic benefit of radiotherapy in CRC and TNBC models.

## Discussion

ENO1 is a multifunctional protein with a ‘main’ function as a glycolytic enzyme in the cytosol and a ‘moonlighting’ function as a plasminogen receptor on the cell surface [50]. Here, we found that ENO1 membrane translocation was regulated by PRMT5-mediated ENO1 methylation via TGFβ1/Smad3 signaling. Surface ENO1 interacts with MCT4 for lactate secretion, which recruits M2 macrophages and promotes an immunosuppressive TME. Targeting surface ENO1 with HuL001, a first-in-class humanized antibody, significantly reduced glycolysis, decreased extracellular lactate accumulation, reprogrammed macrophage polarization and inhibited tumor growth and distant metastasis. Moreover, targeting surface ENO1 significantly increased complete response and delayed tumor regrowth in CRC and TNBC animal models in combination with radiotherapy. The number of antitumoral M1 macrophages and cytotoxic CD8^+^ T cells significantly increased to enhance antitumor immunity and increase the response to radiotherapy (Fig. 7M). These results indicated that targeting surface ENO1 with HuL001 remodeled the tumor microenvironment and provided better therapeutic effects against cancer cells in combination with radiotherapy.

Accumulating evidence indicates that ENO1 mediates cancer cell proliferation, growth, invasion, metastasis, and chemoresistance in multiple types of cancer, including gastric cancer, liver cancer, pancreatic cancer and lung cancer [51–54]. Moreover, there is a positive correlation between ENO1 overexpression and cancer progression in several malignancies [51–54]. These results indicate the need for the development of therapeutic strategies targeting ENO1 for solid cancer treatment. Recent studies have shown that anti-ENO1 DNA vaccines and the small molecule ENOblock that target metabolic processes may be effective in the treatment of PDAC and gastric cancer patients [17, 55, 56]. Additionally, ENO1 externalization of the plasminogen receptor is triggered by the proinflammatory cytokines TGFβ1, CCL2, TNFα, and LPS [24, 57, 58], promoting cancer progression and distant metastasis [59]. In this study, we discovered that surface ENO1 was also associated with cancer progression and poor survival outcomes in CRC, TNBC and lung cancer patients. We found that radiotherapy promoted ENO1 membrane translocation through the TGFβ1/Smad3/PRMT5 pathway [60, 61]. In support of our results, exogenous TGFβ1 triggered PRMT5-depleted SMAD4 and histone H3R2 methylation to promote epithelial‒mesenchymal transition (EMT) for cancer metastasis [60, 61]. In addition, TGFβ1 is also elicited by radiation in cancer cells to hinder the cross-priming of T cells by impairing the antigen-presenting function of dendritic cells and the functional differentiation of T cells into effectors [11]. Therefore, TGFβ1 is the most potent suppressor of the antitumor immune response to limit the therapeutic response to radiotherapy within the TME [12].

Lactate, the metabolic intermediate generated during aerobic glycolysis, is not only utilized as a fuel for growth but also provides acidity to the TME, which promotes the invasion and metastasis of cancer cells [62]. Elevated lactate levels can inhibit the function of a variety of immune cells, such as T cells, natural killer cells and macrophages [10, 63]. The inhibition of MCT4 has been reported as a novel immunotherapeutic strategy to increase phagocytosis and enhance the efficacy of immune checkpoints [8, 10, 64]. Additionally, knockdown of ENO1 or blockade of ENO1 by antibodies can reduce lactate production and release [29, 65]. Consistent with these results, blockade of surface ENO1 significantly inhibited the glycolytic process and decreased lactate release. Furthermore, we discovered that these membranous ENO1 proteins interact with MCT4-dependent lactate secretion for glycolysis. Blockade of ENO1 by HuL001 significantly reprogrammed macrophage polarization for phagocytosis within the tumor microenvironment by decreasing the surface level of MCT4 for lactate secretion. These results suggest that surface ENO1 may stabilize MCT4 to modulate the immunosuppressive tumor microenvironment and polarize M2-like macrophages via lactate. Indeed, Kirk et al. reported that surface expression of MCT4 can be regulated by the plasma membrane glycoprotein CD147, suggesting that the ENO1-MCT4 interaction can stabilize MCT4 surface expression to increase lactate secretion [66]. Additionally, Ray et al. reported that ENO1 acts as a novel immunometabolic target in multiple myeloma (MM). Targeting ENO1 could activate pDCs and increase pDC-induced MM-specific CD8^+^ CTL and NK cell activity to restore anti-MM immunity, enhance MM cytotoxicity, and improve patient outcomes [22]. In support of these findings, our study revealed that targeting surface ENO1 with first-in-class anti-ENO1 antibodies (HuL001) significantly increased the rate of complete response and delayed tumor regrowth in CRC and TNBC animal models in combination with radiotherapy. The number of antitumoral M1 macrophages and cytotoxic CD8^+^ T cells significantly increased to enhance antitumor immunity and increase the response to radiotherapy. These results indicate that targeting surface ENO1 with HuL001 provides better therapeutic effects against cancer cells and eradicates residual tumor cells in combination with radiotherapy. However, the detailed mechanism of ENO1-mediated antitumor immunity remains elusive. Wygrecka et al. reported that surface ENO1 promotes the infiltration of monocytes via its plasminogen‑activating ability [25]. Additionally, ENO1 can destabilize PD-L1 by ubiquitination to sensitize tumor cells and increase T-cell-mediated antitumor immunity in a syngeneic lung cancer mouse model [67]. These results indicate that surface ENO1 may play a complex role in antitumor immunity. However, further studies on the role of surface ENO1 in antitumor immunity are still needed.

Overall, we demonstrated that ENO1 membrane translocation was regulated by PRMT5-mediated ENO1 methylation via TGFβ1/Smad3 signaling. Surface ENO1 interacts with MCT4 for lactate secretion, which recruits M2 macrophages and promotes an immunosuppressive TME. Targeting surface ENO1 significantly delayed tumor regrowth and increased the response to radiotherapy in CRC and TNBC animal models. The number of antitumoral M1 macrophages and cytotoxic CD8^+^ T cells significantly increased to enhance antitumor immunity and increase the response to radiotherapy. These results indicate that targeting surface ENO1 with HuL001 provides better therapeutic effects against cancer cells and eradicates residual tumor cells in combination with radiotherapy.

## Supplementary information

Supplementary information

Western blot Raw data

## Data Availability

The original dataset is available on request from the corresponding author.
