# Supplementary material for: Targeting ENO1 reprograms macrophage polarization to trigger antitumor immunity and improves the therapeutic effect of radiotherapy
Source: Cell Death Dis. 2026 Feb 2;17(1):194. doi: 10.1038/s41419-026-08416-7 (PMC12876979; doi:10.1038/s41419-026-08416-7)
Supplement: Supplementary file 1 — Supplementary information [file 41419_2026_8416_MOESM1_ESM.docx]

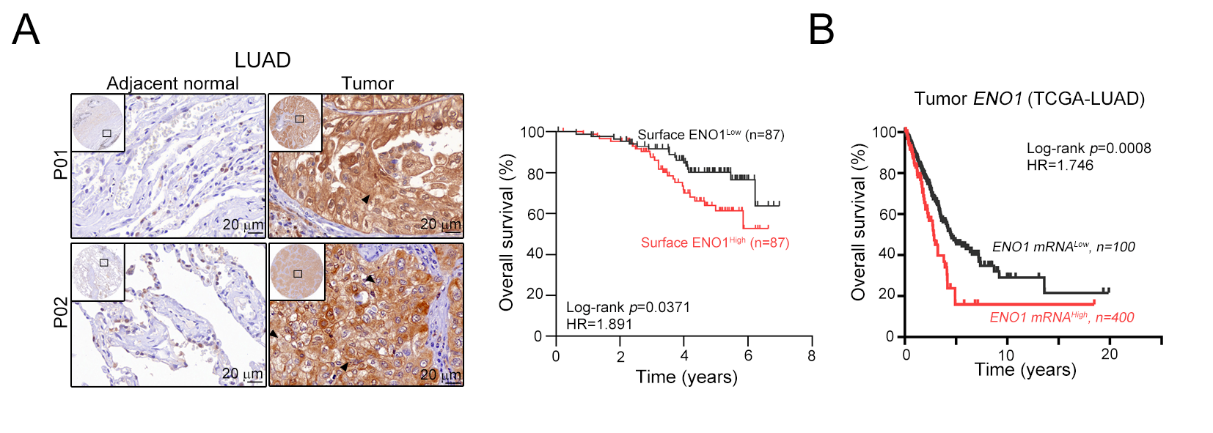


**Supplementary figure 1. High ENO1 expression was associated with poor survival outcome in lung adenocarcinoma (LUAD) patients, which is related to Fig. 1.**

1. The representative images of ENO1 expression in LUAD patients. Patients with high surface ENO1 were associated with poor survival outcome (*n*=174, log-rank *p*=0.0371).
2. High level of *ENO1* mRNA was associated with poor survival outcome in LUAD patients in TCGA database (*n*=500, log-rank *p*=0.0008).


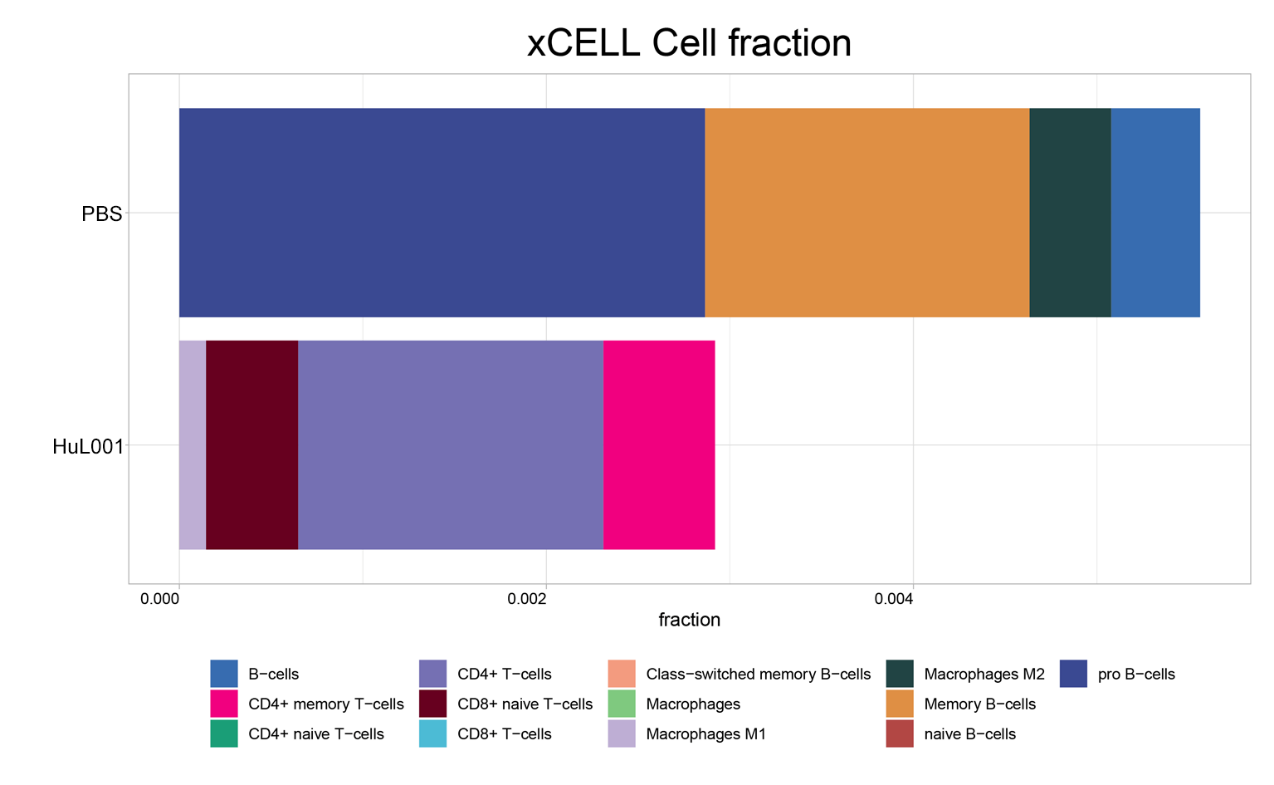


**Supplementary figure 2. The immune cell profiles in the resected tumors from CT26-bearing BALB/c mice, which related to Fig. 3L.**

CT26-bearing BALB/c mice were treated with HuL001 (20 mg/kg, ip) for five times, the tumor tissues were resected for RNA-seq. These transcriptomes were analyzed based on the xCELL cell fraction immune score.


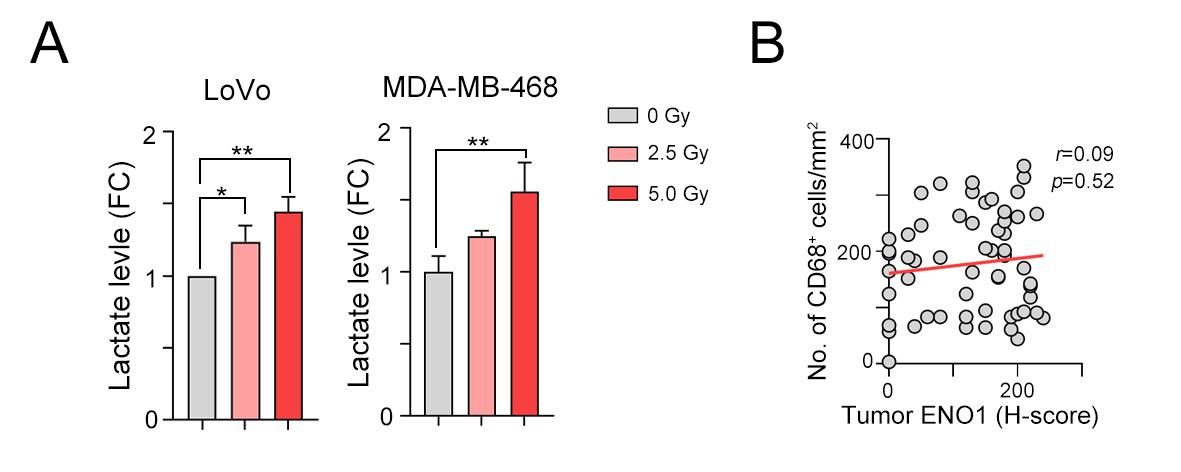


**Supplementary figure 3. The extracellular lactate was increased by radiotherapy, which is related to Fig. 4.**

1. LoVo and MDA-MB-468 cells were irradiated. After 24 h, the conditioned medium was harvested for ELISA analysis.
2. The correlation between CD68 and surface ENO1 was analyzed in the advanced CRC tumor tissues (*n*=56). Pearson correlation analysis (*p*=0.52 and *r*=0.09)


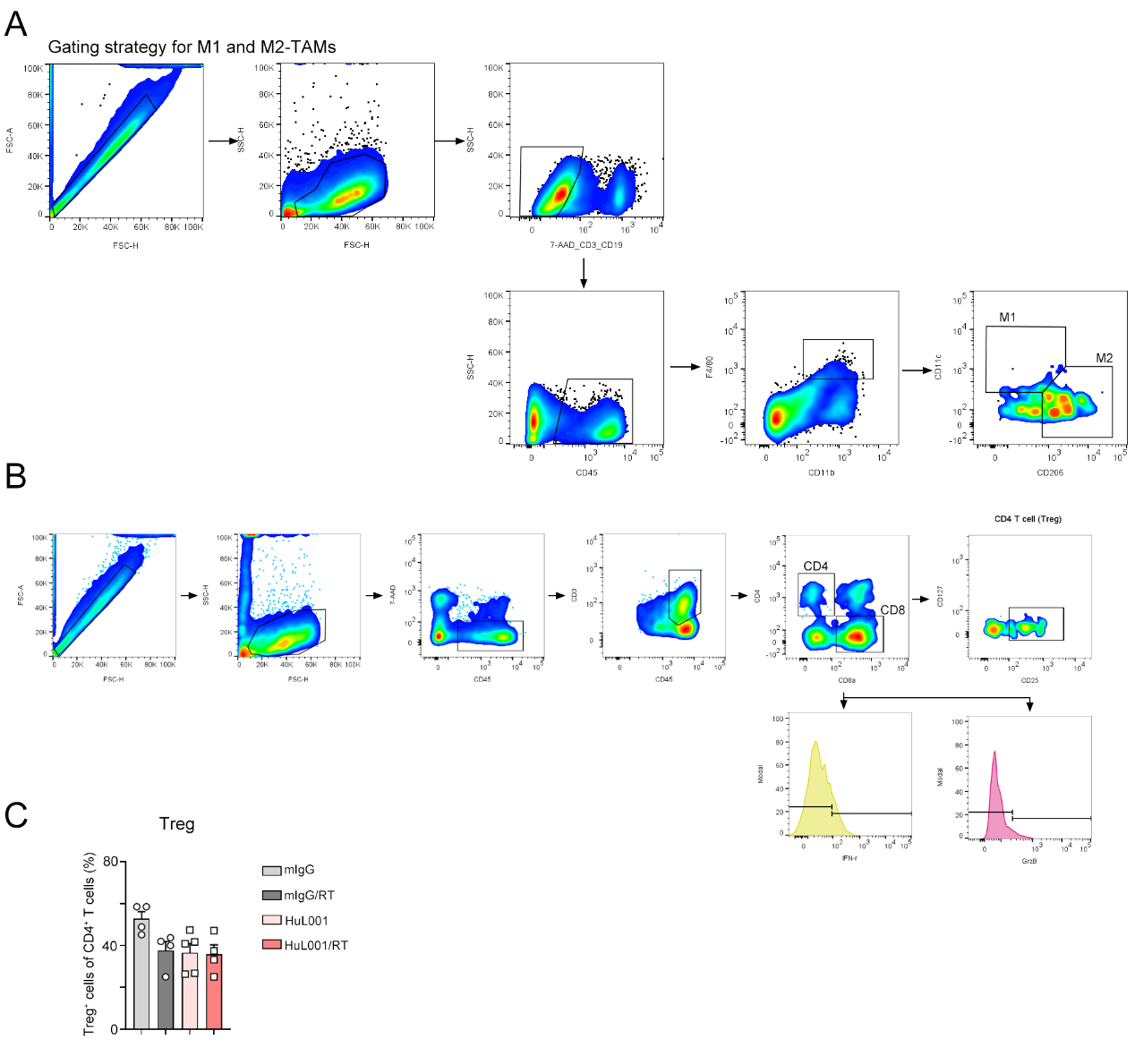


**Supplementary figure 4. The gating strategies for flow cytometry.**

1. The gating strategy for M1 and M2 tumor-associated macrophages.
2. The gating strategy for T cell profiles.
3. The frequency of regulatory T cells CD4 (CD25^+^CD127^-^CD4^+^CD3^+^CD45^+^7AAD^-^) was analyzed by flow cytometry (*n*=3-4). One-Way ANOVA test.
