# Supplementary figures and images for: Targeting ENO1 reprograms macrophage polarization to trigger antitumor immunity and improves the therapeutic effect of radiotherapy
Source: Cell Death Dis. 2026 Feb 2;17(1):194. doi: 10.1038/s41419-026-08416-7 (PMC12876979; doi:10.1038/s41419-026-08416-7)

**A**

Fig. 1A

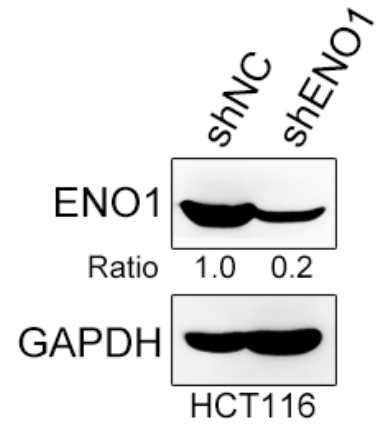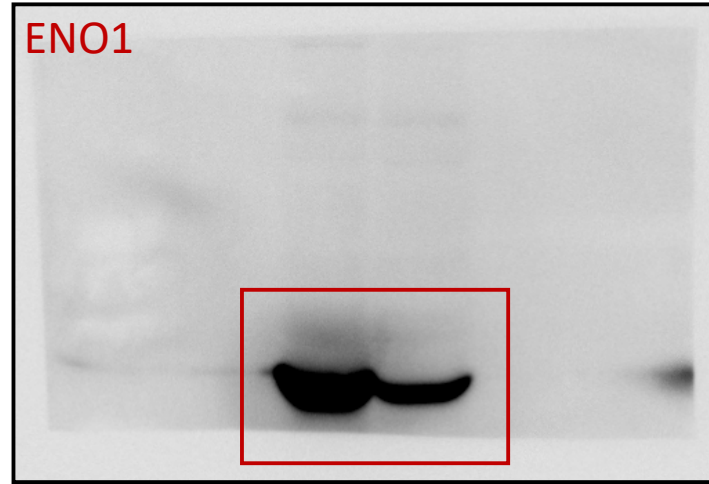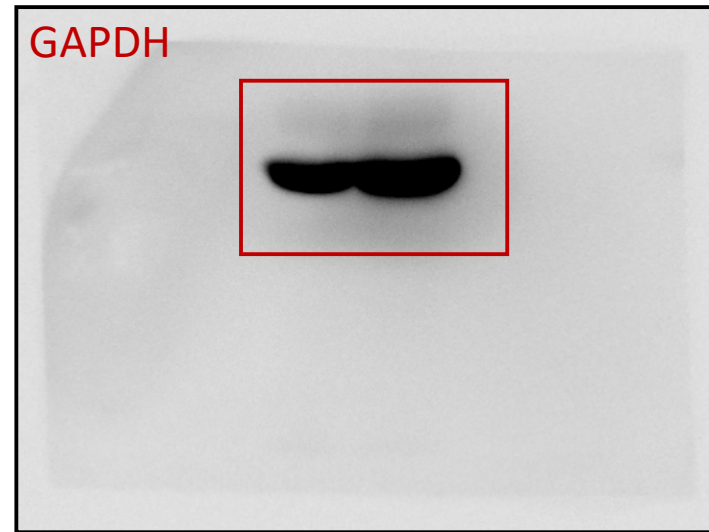

A

Fig. 2A

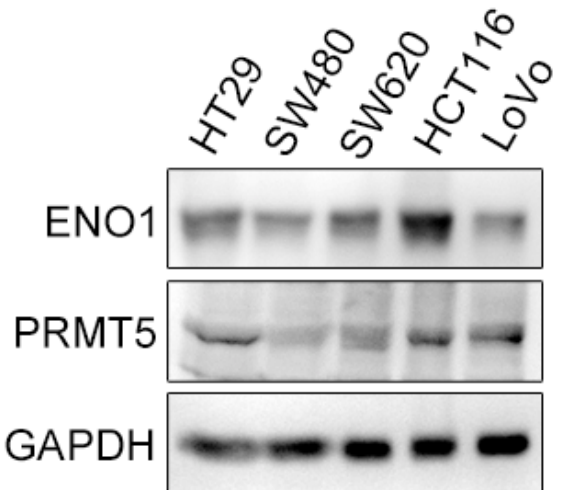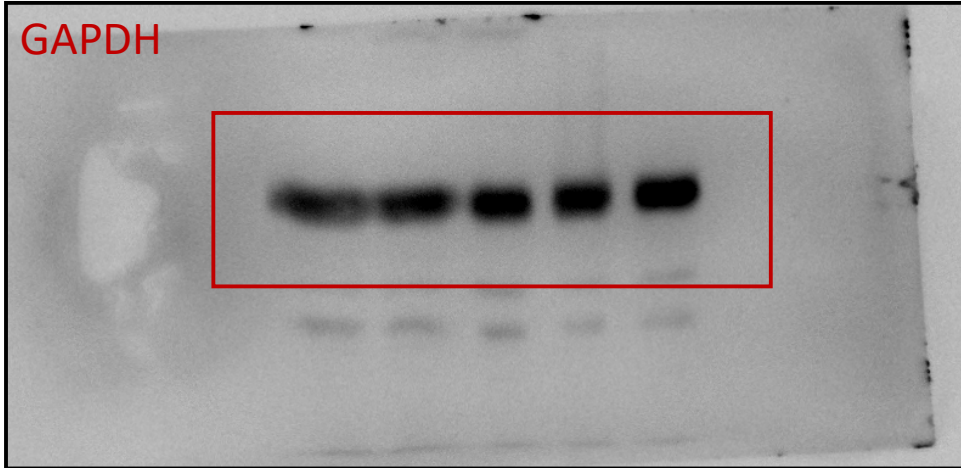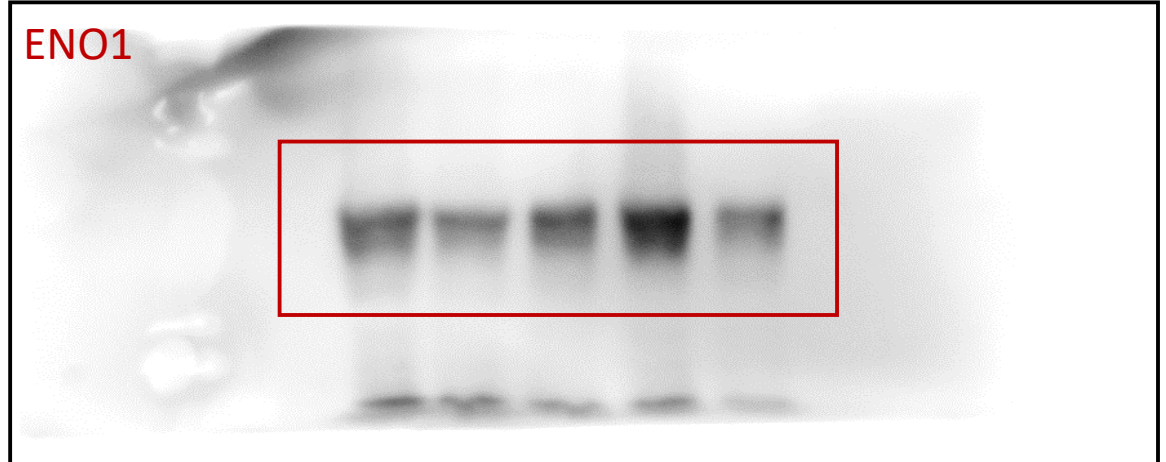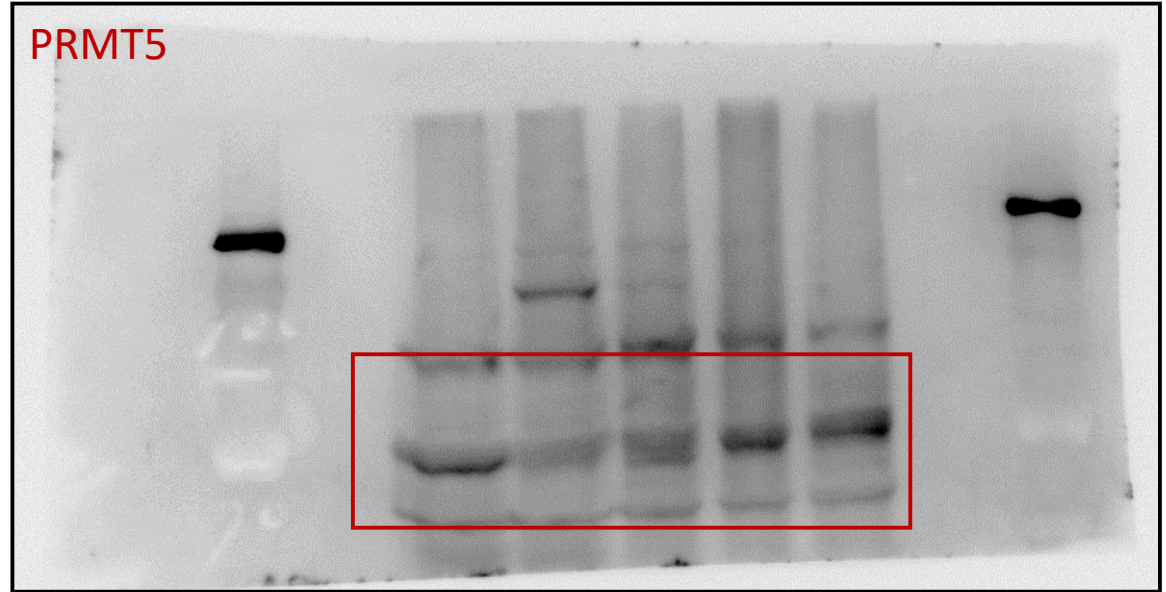

B

Fig. 2B

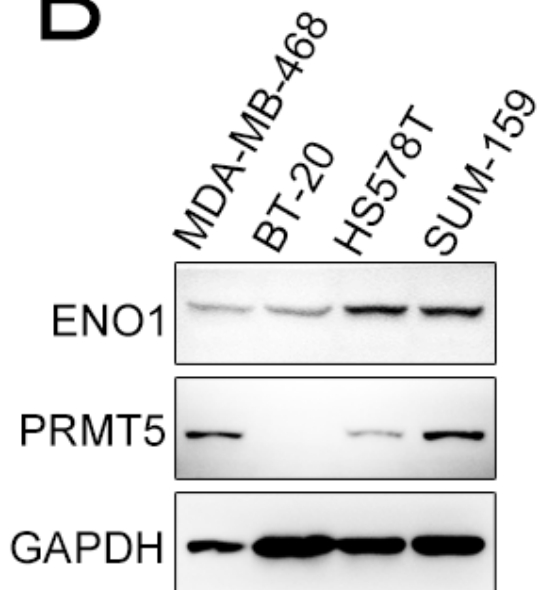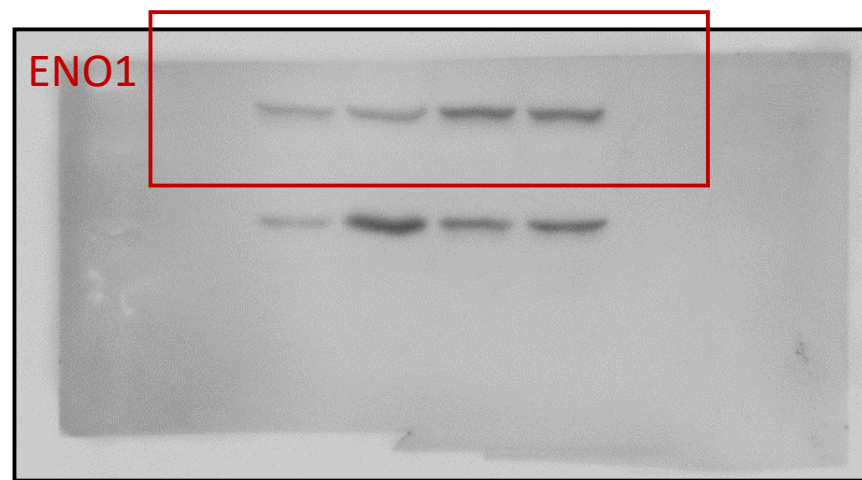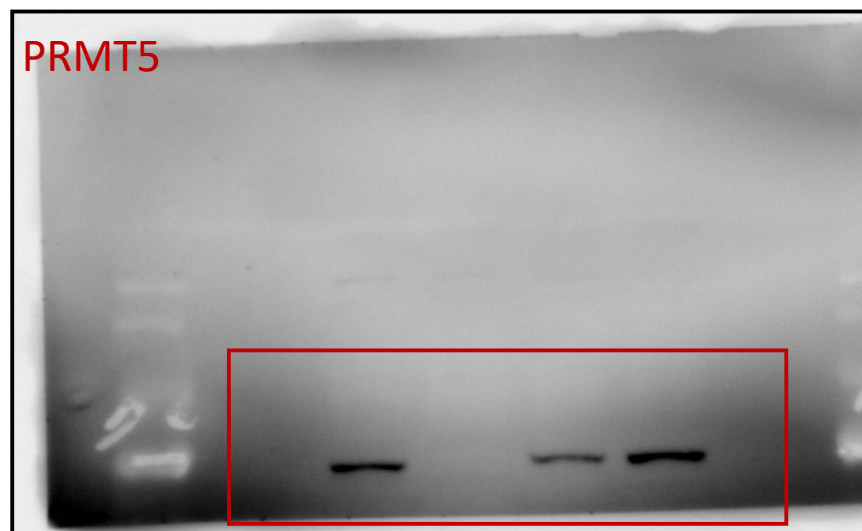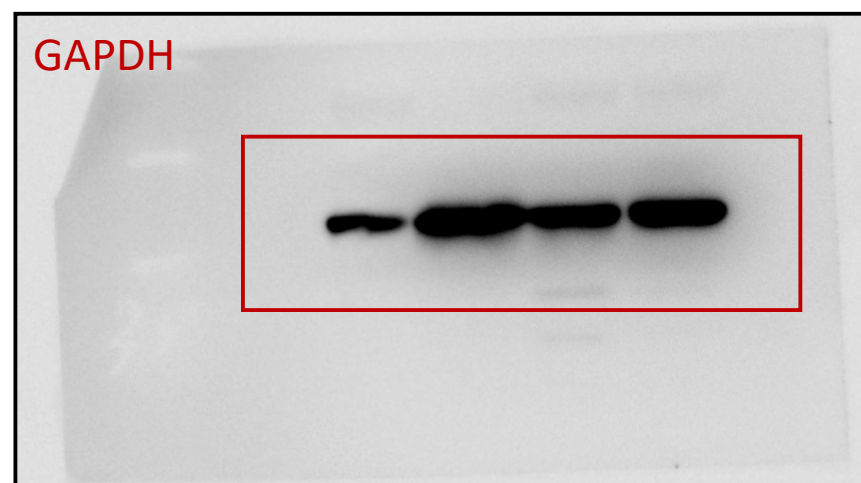

Fig. 2l

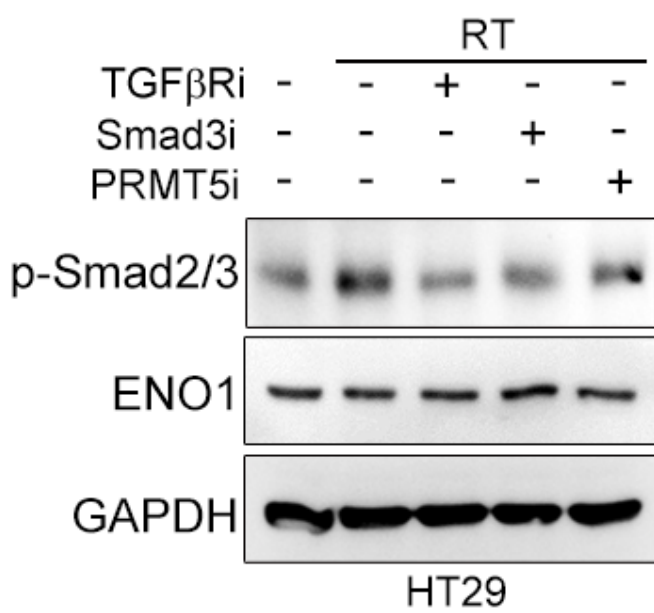

ENO1

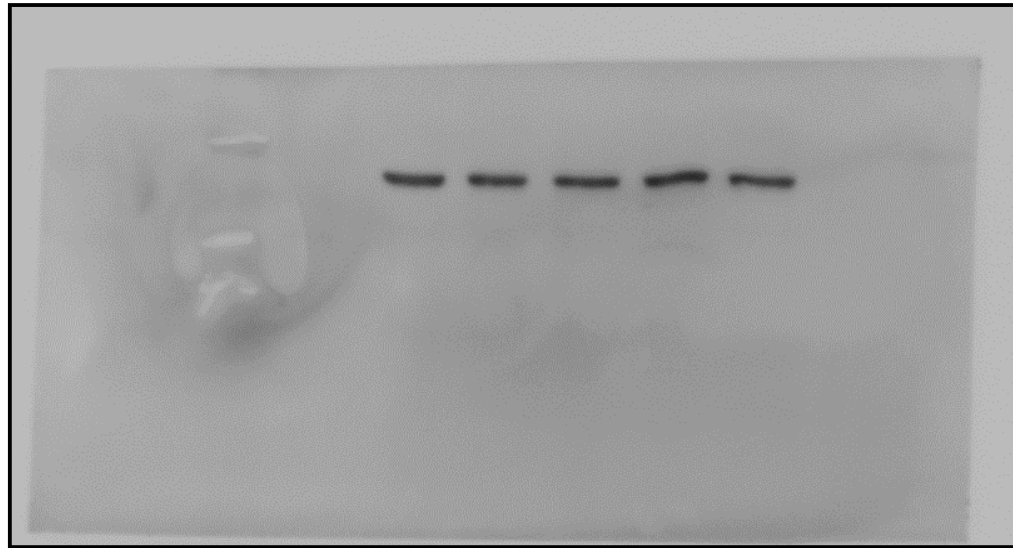

P-SMAD2/3

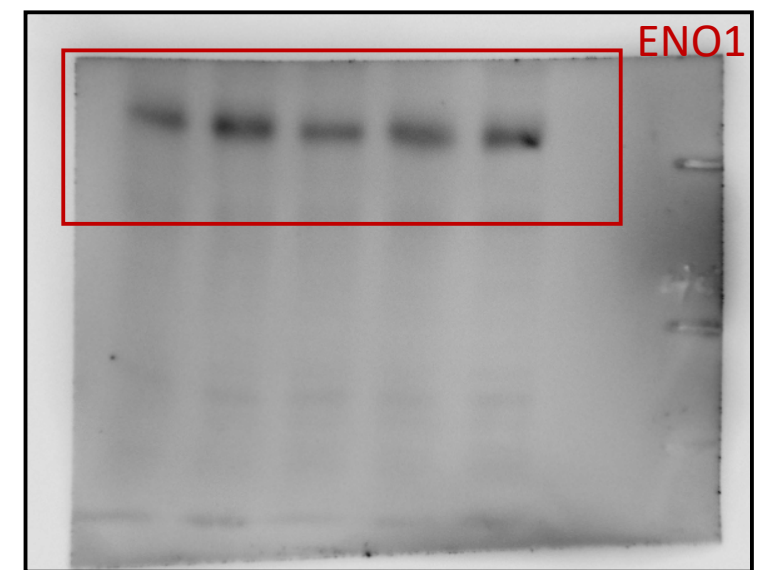

GAPDH

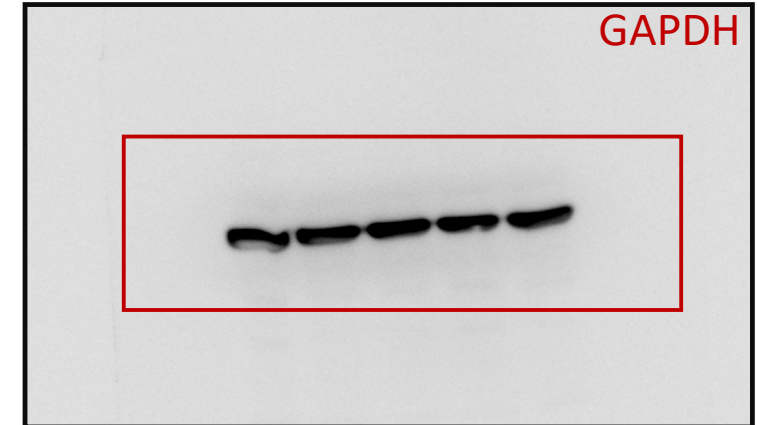

Fig. 2K

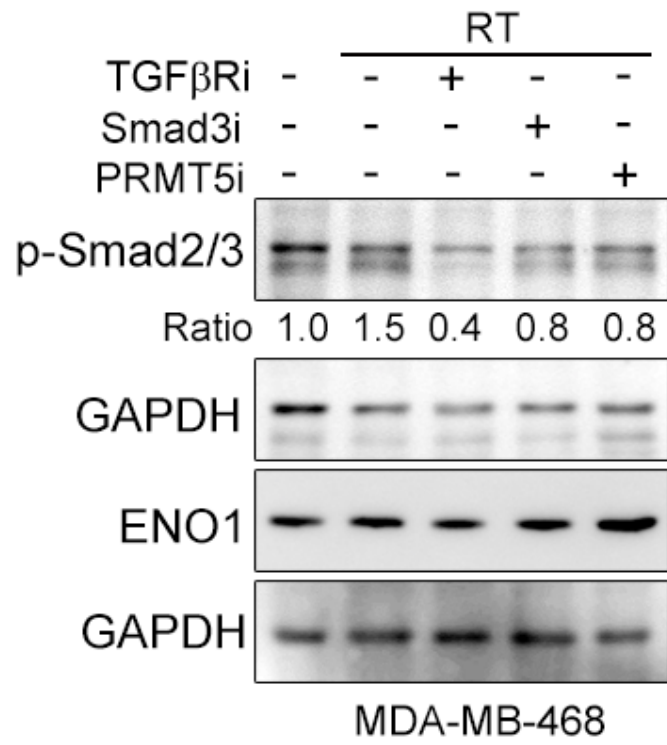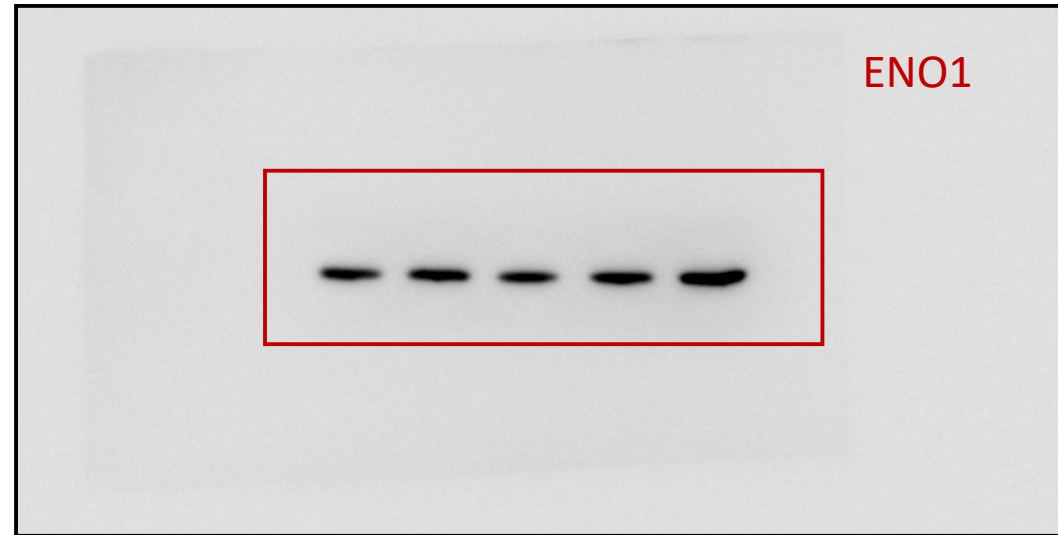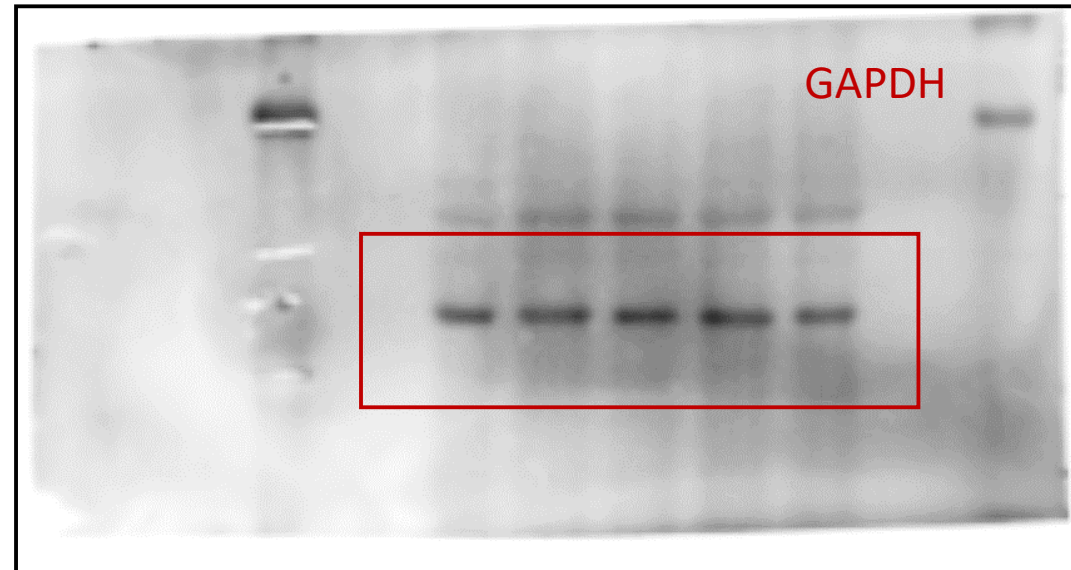

K

Fig. 2K

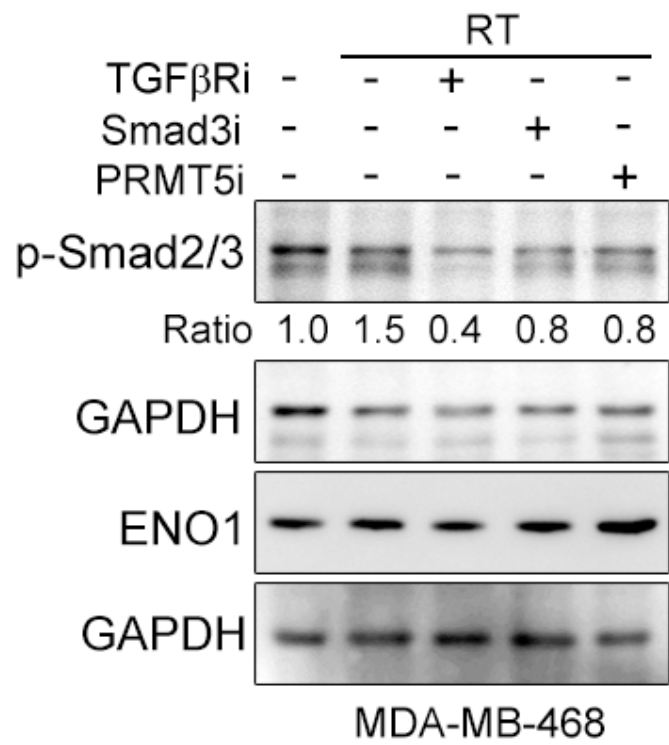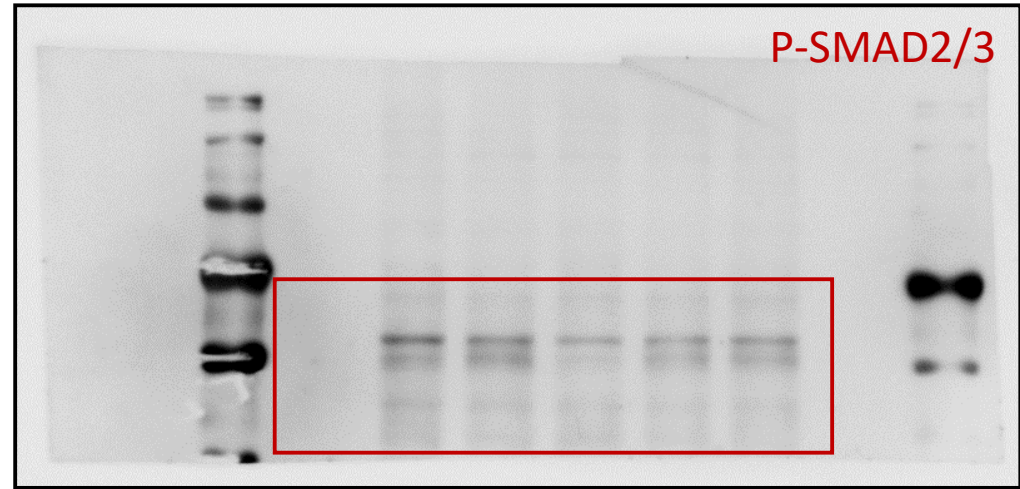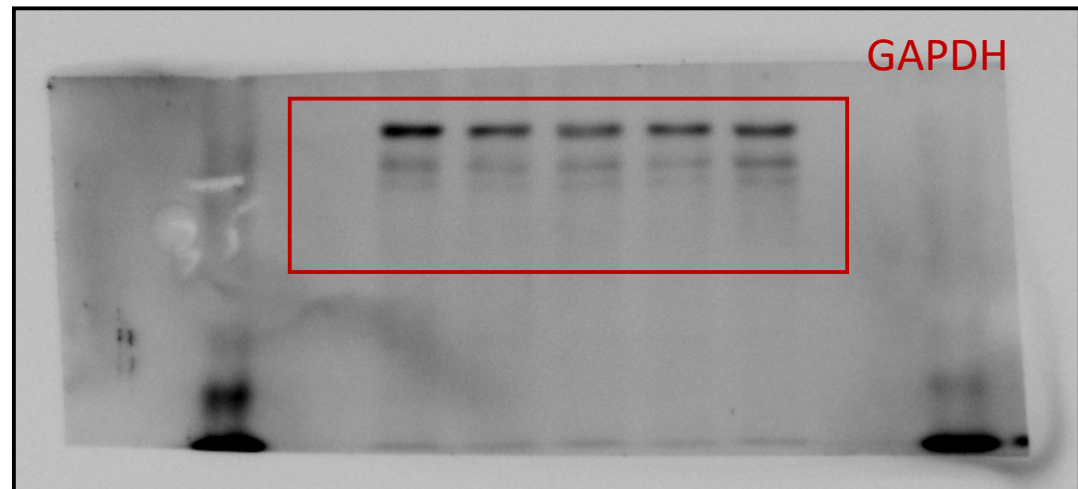

M

Fig. 2M

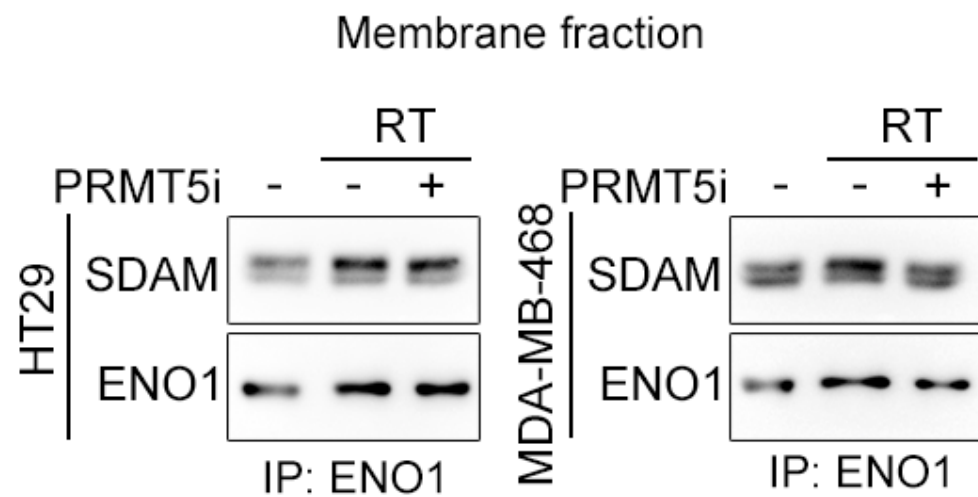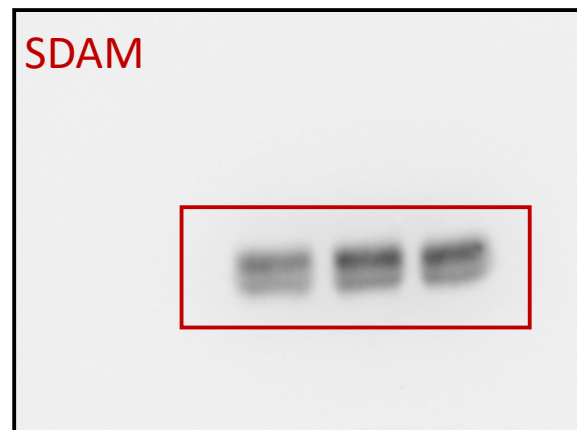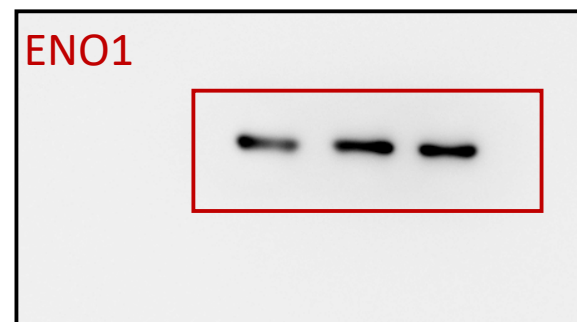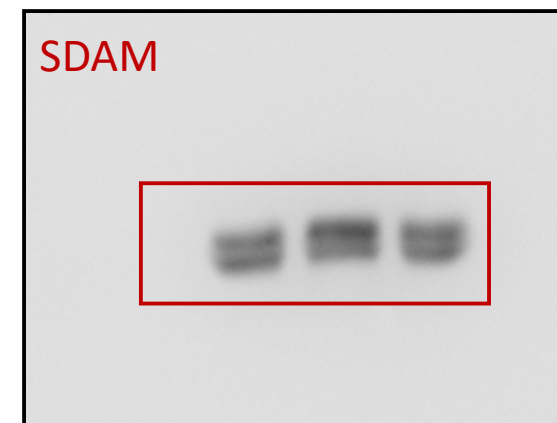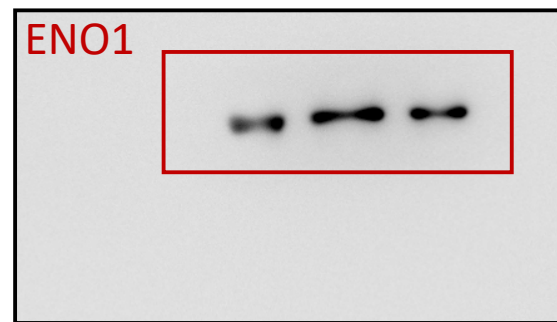

# A

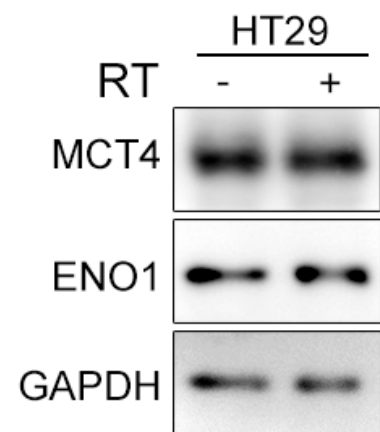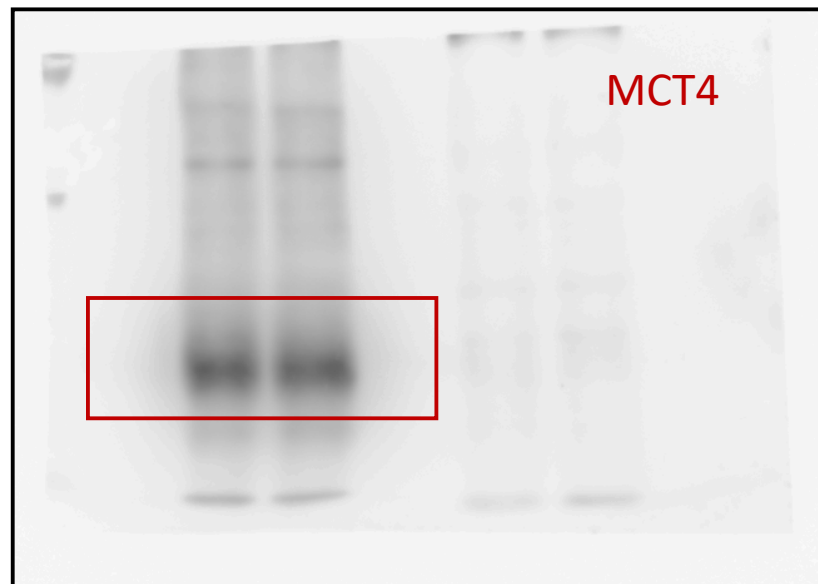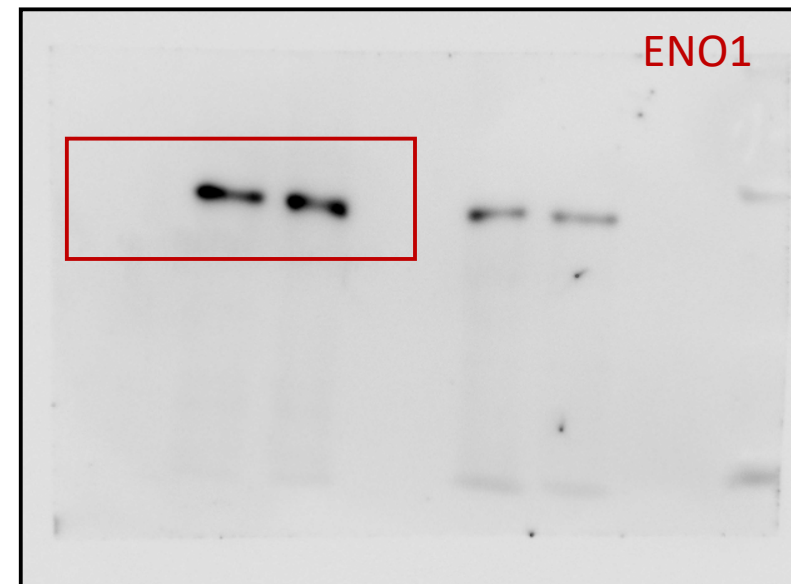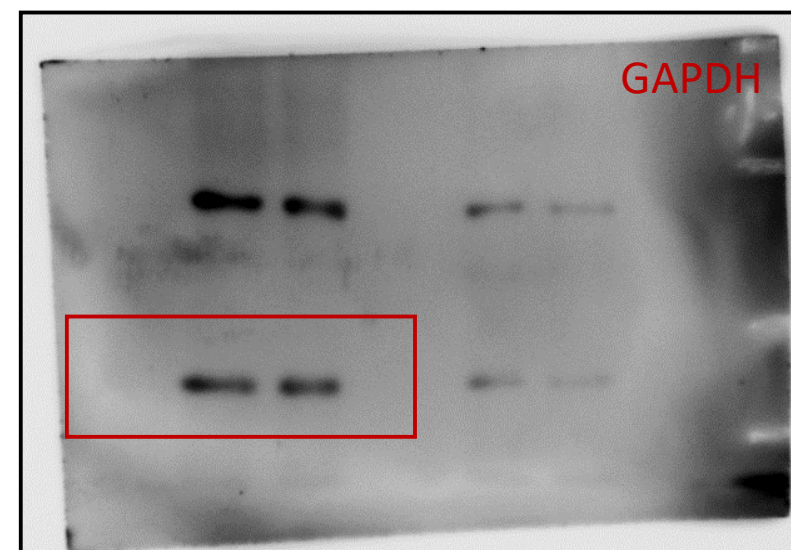

# B

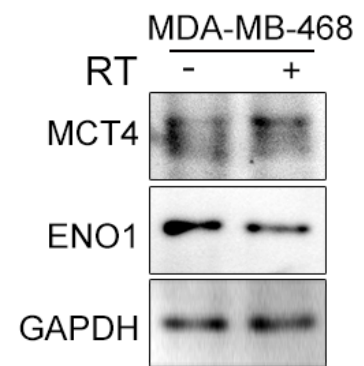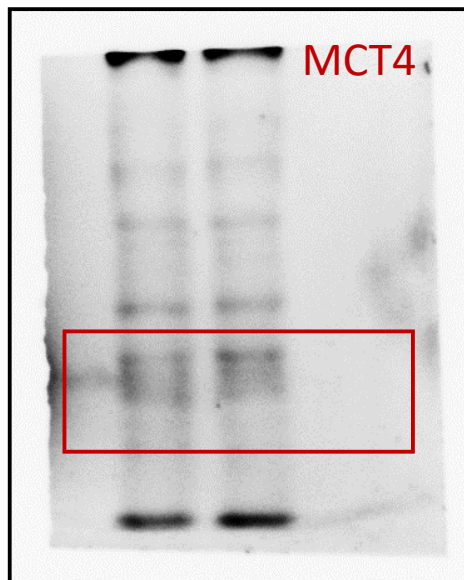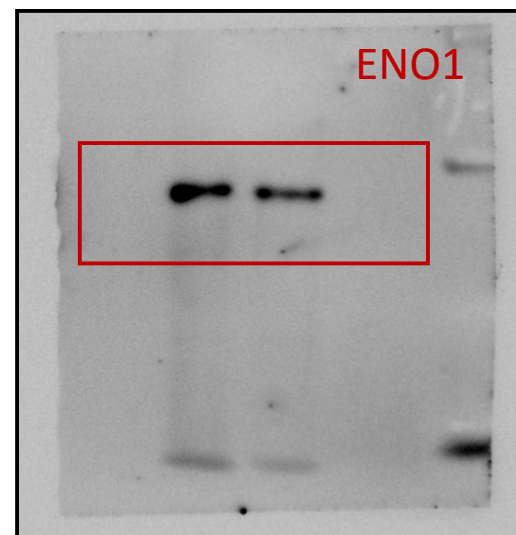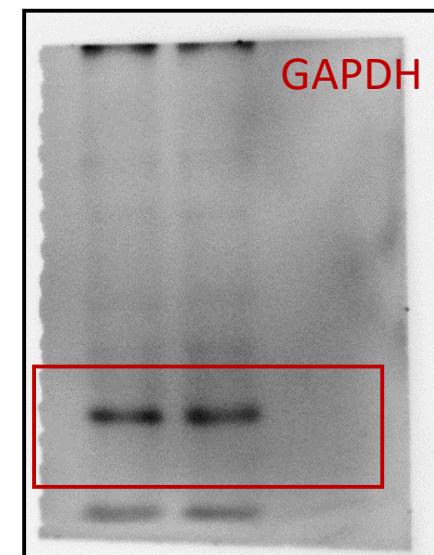

C

HT29

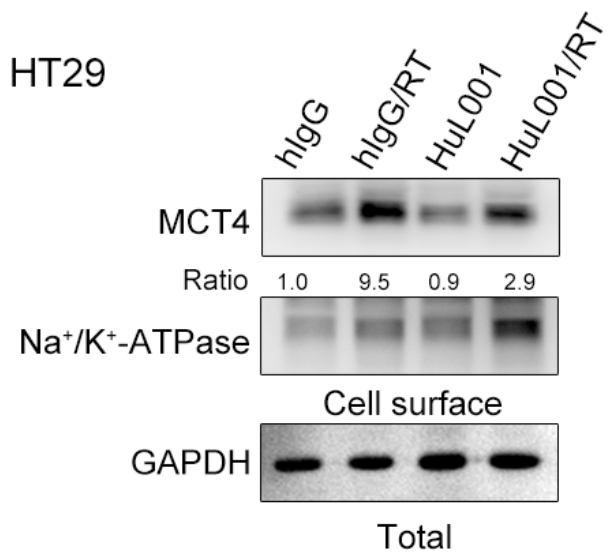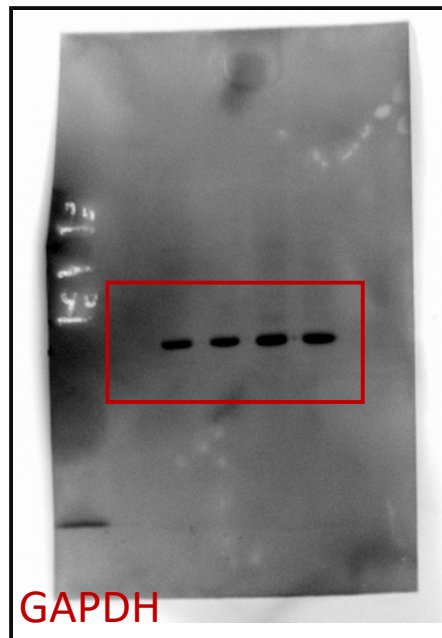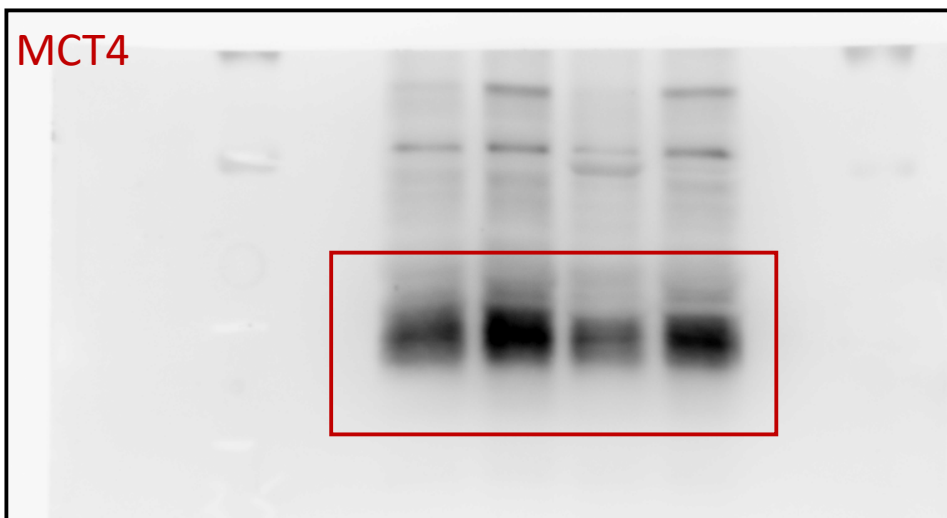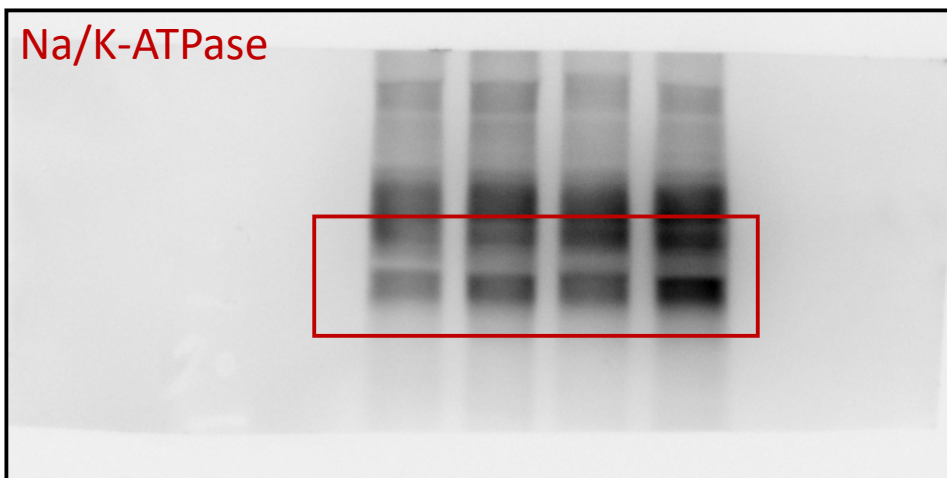

D

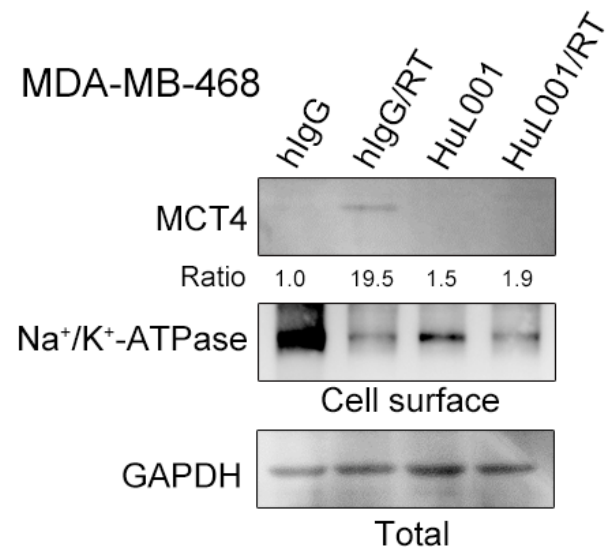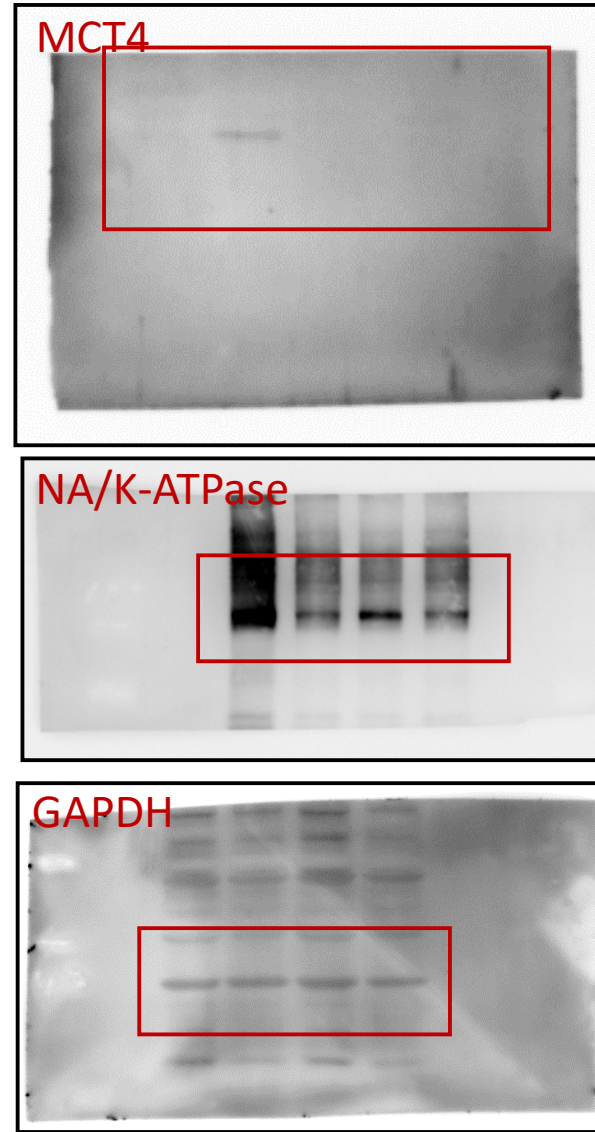

**E**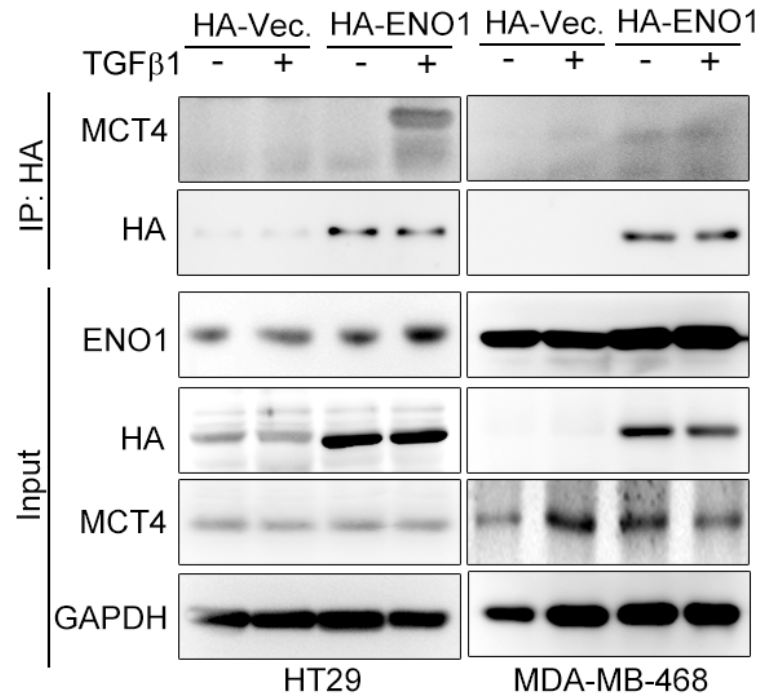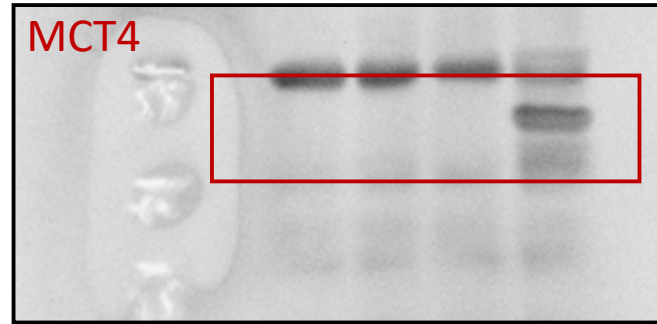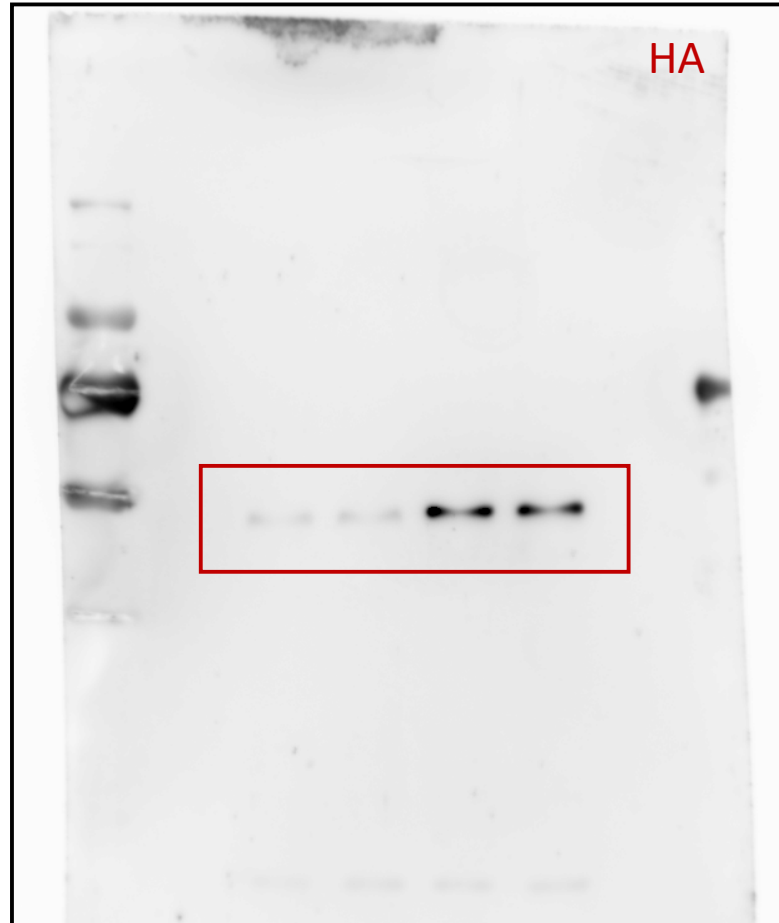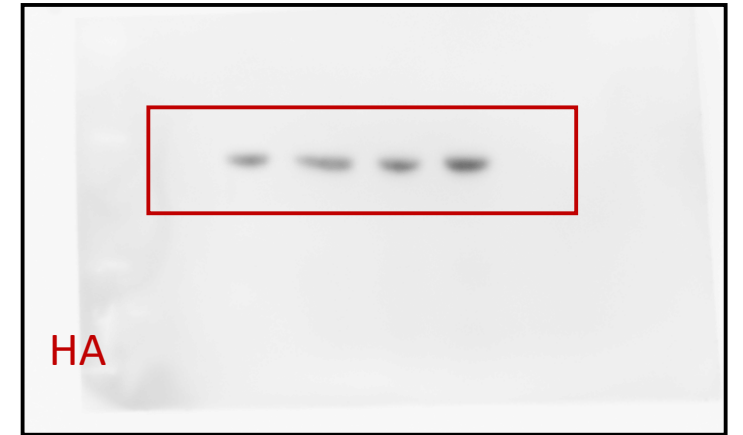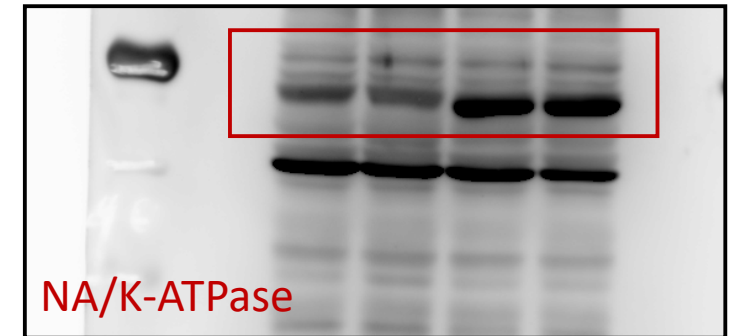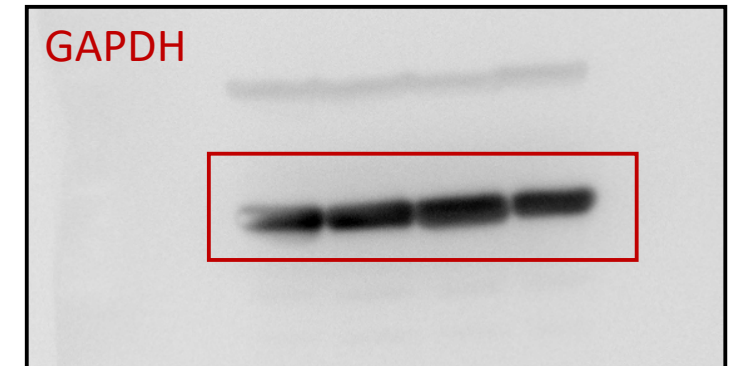

**E**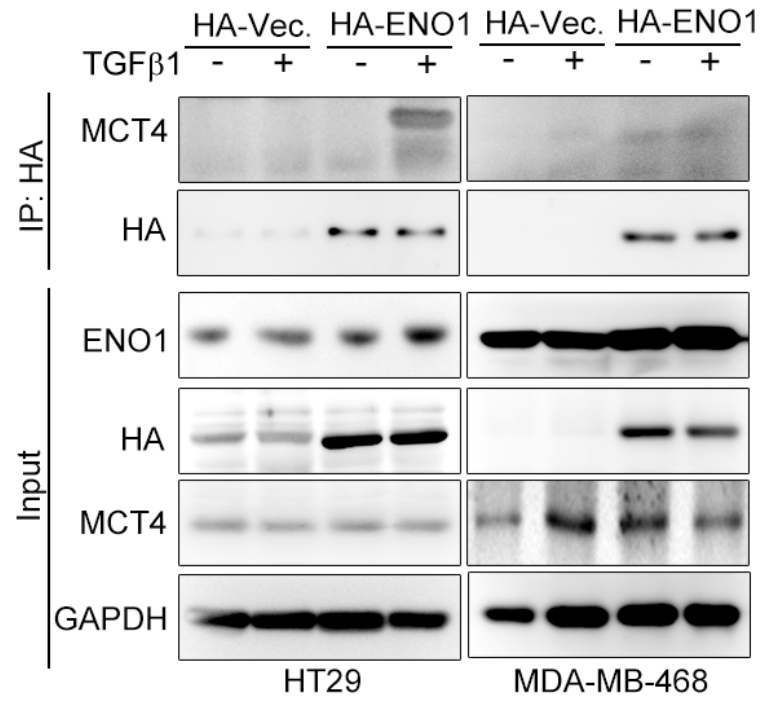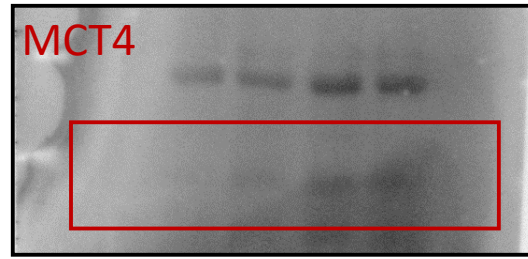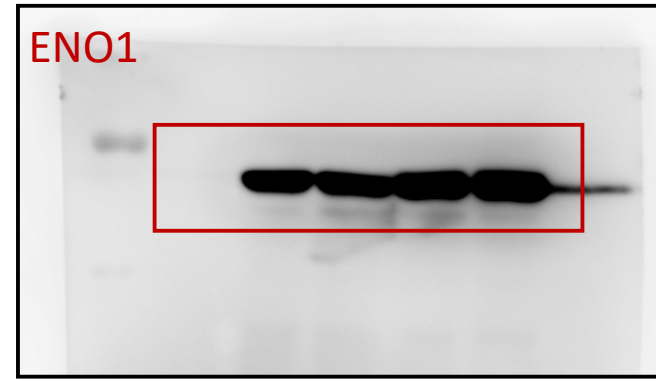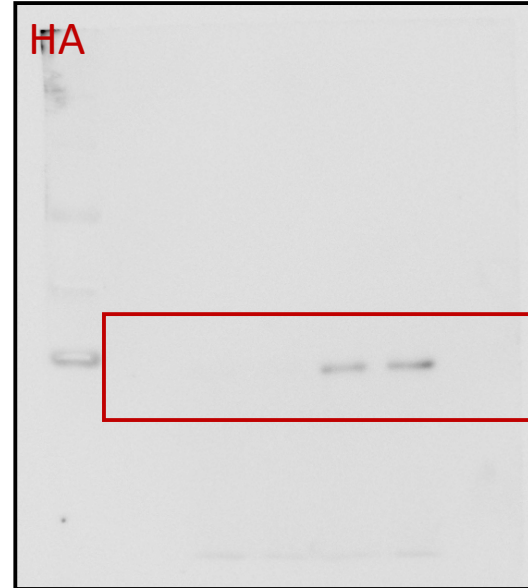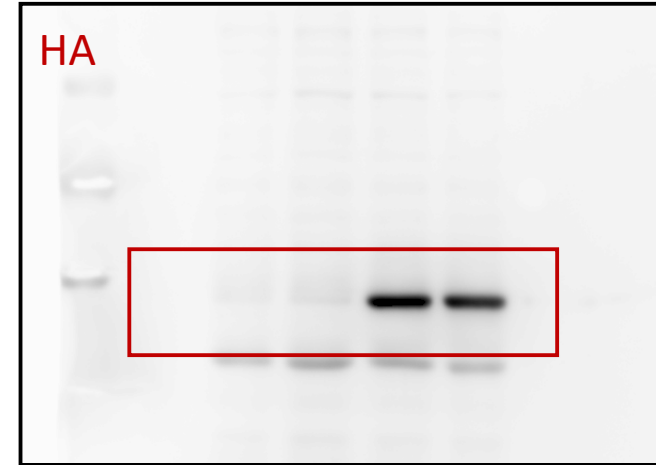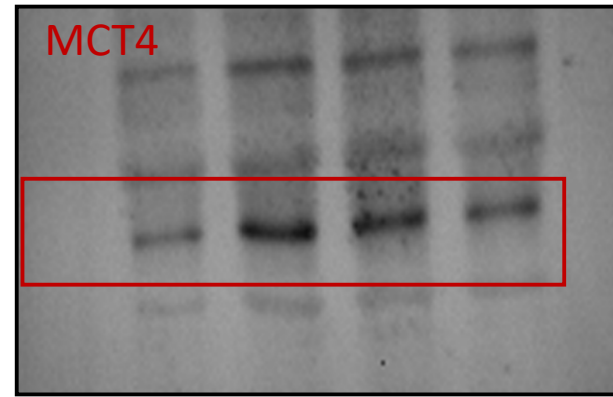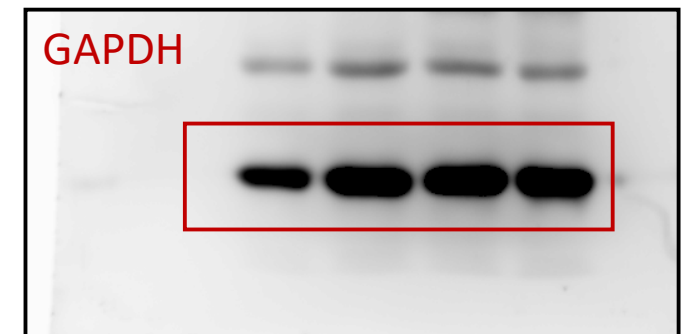

Supplement: Supplementary file 2 — Western blot Raw data [file 41419_2026_8416_MOESM2_ESM.pdf]
